# Tilings with Nonflat Squares: A Characterization

**DOI:** 10.1007/s00032-022-00350-5

**Published:** 2022-03-24

**Authors:** Manuel Friedrich, Manuel Seitz, Ulisse Stefanelli

**Affiliations:** 1 Department of Mathematics, Friedrich-Alexander Universität Erlangen-Nürnberg, Cauerstr. 11, 91058 Erlangen, Germany; 2 Mathematics Münster, University of Münster, Einsteinstr. 62, 48149 Münster, Germany; 3 Faculty of Mathematics, University of Vienna, and Vienna School of Mathematics, Oskar-Morgenstern-Platz 1, 1090 Vienna, Austria; 4 Faculty of Mathematics, University of Vienna, Oskar-Morgenstern-Platz 1, 1090 Vienna, Austria; 5 Vienna Research Platform on Accelerating Photoreaction Discovery, University of Vienna, Währingerstraße 17, 1090 Vienna, Austria; 6 Istituto di Matematica Applicata e Tecnologie Informatiche E. Magenes, via Ferrata 1, 27100 Pavia, Italy

**Keywords:** Nonflat regular square, Configurational energy, Ground state, Characterization, 92E10

## Abstract

Inspired by the modelization of 2D materials systems, we characterize arrangements of identical nonflat squares in 3D. We prove that the fine geometry of such arrangements is completely characterized in terms of patterns of mutual orientations of the squares and that these patterns are periodic and one-dimensional. In contrast to the flat case, the nonflatness of the tiles gives rise to nontrivial geometries, with configurations bending, wrinkling, or even rolling up in one direction.

## Introduction

The serendipitous isolation of graphene in 2004 [25] attracted enormous interest on the physics of 2D materials systems. Driven by their fascinating electronic and mechanical properties [34], research on 2D systems is currently witnessing an exponential growth. Beyond graphene [2, 16], 2D material systems are continuously synthetized and investigated [7, 9, 19, 36] and findings are emerging at an always increasing pace, ranging from fundamental understanding to applications [1].

Free standing 2D material samples are often not flat, but rather present rippling patterns at specific length scales [18]. The origin of such nonflatness is currently debated, one possible explanation being the instability of perfectly flat arrangements at finite temperatures, as predicted by the classical Mermin-Wagner theory [22, 23]. In the case of graphene, ripples have been experimentally observed [20, 24], computationally investigated [12], and analytically assessed [13, 14]. The phenomenon is however not restricted to graphene, and surface rippling has been detected in other 2D systems as well [5, 30]. Understanding the global geometry of 2D materials is of the greatest importance, as flatness is known to influence crucially the electronic, thermal, and mechanical behavior of these systems [8, 10, 33, 35].

**Fig. 1:**
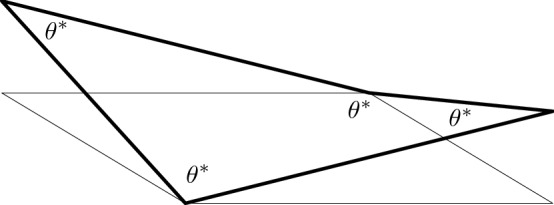
The regular nonflat square

In this paper, we tackle the question of flatness of 2D systems with square symmetry. Our interest is theoretical and our arguments are not tailored to a specific material system. Still, we remark that square-like 2D crystals have been predicted in selenene and tellurene [32]. We formulate the problem in the setting of molecular mechanics [3, 17, 26] by associating to each point configuration a scalar *configurational energy* and focusing on its ground states in the quest for optimal geometries [4, 15]. In the square-symmetric case, each atom has four first neighbors and the topology of the configuration is that of the square lattice ${\mathbb {Z}}^2$ [21]. The configurational energy is assumed to feature both two- and three-body effects [6, 27, 29], depending on bond lengths (distances between atoms) and angles between bonds, respectively. We present conditions ensuring that global minimizers of the configurational energy have all bonds of equal length, all angles formed by bonds to first neighbors of equal amplitude $\theta ^*$, and the four first neighbors of each atom are coplanar. As a result, minimal cycles of four atoms form *regular* squares featuring equal sides and equal angles $\theta ^*$, see Fig. 1. Such identical squares arrange then in an infinite 3D configuration, which under the above provisions we call *admissible* and which we interpret as the actual geometry of the crystal.

The goal of this paper is to classify all admissible configurations, namely all possible 3D arrangements of identical regular squares. In case the squares are flat, namely if $\theta ^*=\pi /2$, the result is straightforward: the only configuration of flat squares where all first neighbors of each atom are coplanar is the plane. In order to tackle genuinely 3D geometries, we hence need to focus on the case $\theta ^*<\pi /2$ instead, which induces nonflatness, as per Fig. 1.

Our main result is a complete characterization of admissible arrangements of identical regular nonflat squares in 3D, see Theorem 2.8. We prove in particular that admissible configurations can bend, wrinkle, and roll in one direction and that such flexural behavior is completely characterized by specifying a suitably defined section of the configuration in the bending direction, see Fig. 4 below. More precisely, one classifies patches of four squares sharing an atom (*4-tiles*) in six different *classes*, in terms of their mutual orientation, see Fig. 6. We prove that just *three* of these classes actually give rise to admissible configurations, that the whole geometry is specified by knowing the pattern of such classes, and that such pattern is periodic.

One can visualize the square in Fig. 1 as (the boundary of) a nonflat tile. Our result can hence be interpreted as a classification of all possible *tilings* with such nonflat tiles under the condition that the four neighbors of each atom are coplanar. The relevance of this coplanarity condition is revealed by considering the limiting flat case. In case tiles are flat and the four neighbors of each atom are coplanar, the only possible tiling is the plane. By dropping the coplanarity requirement, we however allow for tilings ensuing from foldings of the reference square lattice ${\mathbb {Z}}^2$ along a set of parallel coordinate directions. Thus, the coplanarity requirement serves the purpose of excluding the effect of the symmetry of the reference lattice on the onset of nontrivial geometries.

In the case of hexagonal symmetry, the characterization of global arrangements of regular nonflat hexagons has been obtained in [13, 14]. To some extent, the results in this paper for squares are akin to the hexagonal case, for in both cases the arrangement shows some distinguished one-dimensional patterning. Compared with the hexagonal setting, the present square-symmetric case is however much more involved. This is an effect of the different symmetry of the underlying reference lattices. In the square case, arguments require to consider the detailed geometry of patches of up to sixteen neighboring squares, which makes the combinatorial picture much richer.

The paper is organized as follows. Section 2 is devoted to the statement of our main results. The molecular-mechanical model is discussed first and the detailed geometry of ground states is assessed. A first description of admissible configurations is presented in Theorem 2.2. We then introduce the concept of 4-*tile* and of its *type*, collect all possible types and classes, and discuss the possibility of attaching two 4-tiles by analyzing the corresponding boundary, see Lemma 2.6. This eventually paves the way to the statement of our main result, namely the characterization of Theorem 2.8. Section 3 is entirely devoted to the proof of the main result, hinging both on combinatorial and geometrical arguments. Some proofs are postponed to the Appendix in order to enhance the readability of the arguments.

## The Setting and Main Results

### Ground States of Configurational Energies

We focus on three-dimensional deformations $y:{\mathbb {Z}}^2 \rightarrow {\mathbb {R}}^3$, defined on the two-dimensional *reference lattice* ${\mathbb {Z}}^2$. For any open subset $\Omega \subset {\mathbb {R}}^2$ we define the *configurational energy* of a deformation on $\Omega$ by

2.1 $$\begin{aligned} E(y,\Omega ):= & {} \frac{1}{2}\sum _{(x,x') \in N_1(\Omega )} v_2\big (|y(x) - y(x')|\big ) + \frac{1}{2} \sum _{(x,x') \in N_2(\Omega )} v_2\big (|y(x) - y(x')|\big )\nonumber \\&+ \frac{1}{2}\sum _{(x,x',x'') \in T(\Omega )} v_3\big (\measuredangle y(x)\, y(x') \, y(x'')\big ), \end{aligned}$$

where

2.2 $$\begin{aligned} N_1(\Omega ):=\big \{ (x,x'):x,x' \in {\mathbb {Z}}^2, \, x \in \Omega , \, x' \in {\overline{\Omega }}, \, |x-x'|=1\big \} \end{aligned}$$

denotes the set of *nearest-neighbors* and

2.3 $$\begin{aligned} \begin{aligned} N_2(\Omega )&:=\lbrace (x,x') :x,x' \in {\mathbb {Z}}^2 \cap {\overline{\Omega }}, \, |x-x'|=\sqrt{2}; \\&\quad \quad (x-x')\cdot e_1>0 \,\text {if}\, x\in \partial \Omega \,\text {or}\, x'\in \partial \Omega \rbrace \end{aligned} \end{aligned}$$

is the set of *closest next-to-nearest-neighbors*. Moreover, by $\measuredangle y(x)\, y(x') \, y(x'')$ we denote the *bond angle* in $[0,\pi ]$ at $y(x')$ formed by the the vectors $y(x)-y(x')$ and $y(x'')- y(x')$, where the set of *triplets* $T(\Omega )$ is defined by

2.4 $$\begin{aligned} T(\Omega ):=\lbrace (x,x',x''):(x',x) \in N_1(\Omega ), (x',x'') \in N_1(\Omega ), x \ne x'' \rbrace . \end{aligned}$$

The factor 1/2 reflects the fact that *bonds* $\lbrace y(x), y(x') \rbrace$, $(x,x') \in N_1(\Omega ) \cup N_2(\Omega )$, and bond angles $\measuredangle y(x)\, y(x') \, y(x'')$ appear twice in the corresponding sums. Let us point out that in order to take surface effects at $\partial \Omega$ properly into account, bonds $\lbrace y(x), y(x') \rbrace$ are only counted once if $\{ x, x'\} \in N_1(\Omega )$ and either $x\in \partial \Omega$ or $x' \in \partial \Omega$, or if $\{x,x'\} \in N_2(\Omega )$ and $x \in \partial \Omega$ or $x' \in \partial \Omega$. Bonds where $\{x,x'\} \in N_1(\Omega )$ with $x \in \partial \Omega$ and $x'\in \partial \Omega$ are not counted at all. This asymmetry of counting bonds is motivated by the specific choice of the cell energy, see Sect. A.5.

We assume the *two-body* interaction potential $v_2:{\mathbb {R}}^+\rightarrow [-1,\infty )$ to be of short-range repulsive and long-range attractive type. In particular, we assume that $v_2$ is continuous and attains its minimum value only at 1 with $v_2(1) = -1$. Moreover, we suppose that $v_2$ is decreasing on (0, 1), increasing on $[1,\infty )$, and that $v_2$ is continuously differentiable on (1, 2] with $v_2' >0$ on (1, 2]. The *three-body* interaction density $v_3:[0,\pi ] \rightarrow [0,\infty )$ is assumed to be strictly convex and smooth, with ${v}_3(\pi ) = 0$.

In the following, we will be interested in minimizing the energy of a configuration on the *whole* reference lattice. To this end, we define the *normalized energy* of $y:{\mathbb {Z}}^2 \rightarrow {\mathbb {R}}^3$ by

2.5 $$\begin{aligned} E(y) = \sup _{m \in {\mathbb {N}}} \frac{1}{(2m-1)^2} E(y,Q_m), \end{aligned}$$

where $Q_m \subset {\mathbb {R}}^2$ is the open square centered at 0 with sidelength 2*m*. A deformation is called a *ground state* if it minimizes the energy *E*.

For a fine characterization of the minimizers, some additional qualification on $v_2$ and $v_3$ will be needed. More precisely, we suppose that there exist small parameters $\eta , \varepsilon >0$ such that

2.6 $$\begin{aligned}&v_2(1-\eta ) > 3 + 4 v_2(\sqrt{2}) +8v_3(\pi /2), \end{aligned}$$

2.7 $$\begin{aligned}&v_2(1+\eta ) > -1 + 4 v_2(\sqrt{2}) - 4v_2(\sqrt{2}(1-\eta )^2) +8v_3(\pi /2), \end{aligned}$$

2.8 $$\begin{aligned}&v_3(\theta ) > 2 + 2 v_2(\sqrt{2}) +4v_3(\pi /2) \text { if } \theta \le \pi /2 - \eta , \end{aligned}$$

2.9 $$\begin{aligned}&( \ell _1,\ell _{2} ,\theta ) \mapsto \dfrac{1}{4} v_2( \ell _1) + \dfrac{1}{4} v_2( \ell _2) + v_2 \left( \left( \ell _1^2+ \ell _2^2 - 2 \ell _1 \ell _2 \cos \theta \right) ^{1/2} \right) + v_3(\theta ) \nonumber \\&\quad \quad \text {strictly convex on}\, [1-\eta ,1+\eta ]^2\times [\pi /2- \eta ,\pi ]\,\text { and }\nonumber \\&\quad \quad \text {strongly convex for }\,\theta \in [\pi /2- \eta ,\pi /2+3\eta ], \end{aligned}$$

2.10 $$\begin{aligned}&|v_3|,|v_3'| \le \varepsilon \text { in a neighborhood of}\, \pi , \end{aligned}$$

2.11 $$\begin{aligned}&0< -2\sqrt{2} \sqrt{1-\cos \theta } v_3'(\theta ) < \ell \sin \theta \, v_2'\big (\sqrt{2}\ell \sqrt{1-\cos \theta }\big ) \nonumber \\&\quad \quad \text { for }\, \ell \in [1-\eta , 1] \,\text { and }\, \theta \in [\pi /2-\eta ,\pi ]. \end{aligned}$$

Properties (2.6)–(2.7) entail that first-neighbor bond lengths range between $1-\eta$ and $1+\eta$, whereas (2.8) ensures that bond angles are not significantly smaller than $\pi /2$. Eventually, assumptions (2.9)–(2.11) yield that the contributions of first and second neighbors are strong enough to induce local geometric symmetry of ground states, i.e., bonds and bond angles will be constant, see (2.12)–(2.14) below.

Note that the assumptions (2.6)–(2.11) are compatible with a choice of a density $v_2$ growing sufficiently fast out of its minimum. In particular, the quantitative Lennard–Jones-like case of Theil [28] (see also [11, 31]) can be reconciled with assumptions (2.6)–(2.7), upon suitably choosing densities and parameters. Let us however remark that the specific form of (2.6)–(2.11) is here chosen for the sake of definiteness and simplicity. Indeed, these assumptions could be weakened, at the expense of additional notational intricacies. Under the above assumptions we have the following result, where we set $N_1 :=N_1({\mathbb {R}}^2)$, $N_2 :=N_2({\mathbb {R}}^2)$, and $T :=T({\mathbb {R}}^2)$ (see (2.2)–(2.4)).

#### Proposition 2.1

(Ground states). Let $v_2$ and $v_3$ be the above-introduced two- and three-body-potentials satisfying assumptions (2.6)–(2.11). For $\eta$ small enough and $\varepsilon = \varepsilon (\eta )$ small enough there exist $\ell \le 1$, $\theta <\pi /2$, and $\delta _\theta < \pi$ only depending on $v_2$ and $v_3$ such that a deformation $y:{\mathbb {Z}}^2 \rightarrow {\mathbb {R}}^3$ is a ground state of the energy *E* if and only if *y* satisfies

2.12 $$\begin{aligned} |y(x) - y(x')| =\ell \quad \text {for all}\, (x,x') \in N_1, \end{aligned}$$

and2.13as well as2.14

Here, the conditions $(x,x'') \in N_2$ and $(x,x'') \notin N_2$ correspond to the case that the vectors $x-x'$ and $x''-x'$ form an angle $\pi /2$ or $\pi$, respectively, in the reference lattice. We will see later that $\delta _\theta$ is uniquely determined by $\theta$ due to a geometric compatibility condition, see Lemma 2.4 below.

The proof of Proposition 2.1 is similar to the one in [13, Proposition 3.1] and is postponed to Appendix A.5. At this stage, let us just comment on the effect of condition $v_2' > 0$ in a neighborhood of $\sqrt{2}$, see (2.11), which guarantees that $\theta$ is strictly smaller than $\pi /2$. Indeed if $v_2' = 0$ in a neighborhood of $\sqrt{2}$, we would obtain $\ell = 1$ and $\theta = \pi /2$, i.e., $y({\mathbb {Z}}^2)$ would coincide with ${\mathbb {Z}}^2$ up to isometries. For $\theta <\pi /2$ instead, ground states exhibit interesting nontrivial geometries. The aim of this paper is precisely that of characterizing these nontrivial geometries.

### Necessary Conditions for Admissibility

Deformations $y:{\mathbb {Z}}^2 \rightarrow {\mathbb {R}}^3$ satisfying the conditions (2.12)–(2.14) are called *admissible*. Without restriction, we suppose for notational convenience that $\ell =1$. Indeed, this can be achieved by replacing *y* by $\frac{1}{\ell }y$ without effecting the geometry of admissible configurations.

**Fig. 2:**
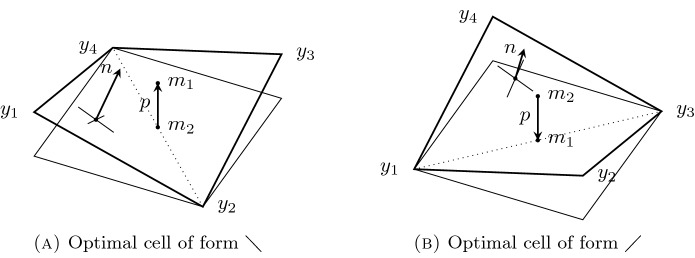
The two optimal cells, defined via the vector *p* and the normal vector of one face. For the sake of illustration, they are positioned in such a way that *p* is parallel to $e_3$, which is why here $y_1 \cdot e_3 = y_3 \cdot e_3$ and $y_2 \cdot e_3 = y_4 \cdot e_3$. In symbols, we indicate optimal cells with $\diagup$ or $\diagdown$, according to the direction of the lower diagonal

Obviously, conditions (2.12)–(2.13) constrain the local geometry of configurations: let $\{x_1, x_2, x_3, x_4\}$ be a simple cycle in ${\mathbb {Z}}^2$, called a *reference cell*, where here and in the following the labeling is counterclockwise and counted modulo 4. The image via *y* is the simple cycle $\{y_1, y_2, y_3, y_4\}$, where $y_i = y(x_i)$, called an *optimal cell*. Since $\theta < \pi /2$ from (2.13), optimal cells are not flat. Indeed, the sum of interior angles is strictly less then $2\pi$, i.e., $\sum _{i = 1}^{4} \measuredangle y_{i-1} \, y_i \, y_{i+1} = 4 \theta < 2 \pi$, see also Fig. 2.

The kink of an optimal cell can equivalently be visualized as occurring along the diagonal $$x_3 - x_1$$ or along the diagonal $$x_4 - x_2$$ of the corresponding reference cell. We set $$m_1 := (y_1 + y_3)/2$$ and $$m_2 := (y_2 + y_4)/2$$ and define $$p := m_1 - m_2$$. Let *n* be the normal vector of the triangle formed by $$ y_1$$, $$y_2$$, and $$y_4$$, in direction $$(y_2 - y_1) \times (y_4-y_2)$$. Then, we say that the optimal cell is of * form* $$\diagdown $$ if $$p \cdot n > 0$$ and of * form* $$\diagup $$ if $$p \cdot n < 0$$, see Fig. 2. An optimal cell of any form can be transformed into a cell of the other form simply via a rotation by $$\pi /2$$ along the vector *p* or via a reflection with respect to the plane with normal *p*.

Our goal is to provide a complete characterization of admissible configurations. In a first step, we will present necessary conditions for admissibility in terms of *optimal cells*. To obtain a complete characterization, we will subsequently present a refined formulation in terms of so-called 4-*tiles*, namely, $2\times 2$ groups of optimal cells, see Sect. 2.5. To state our first main result, we need to introduce some further notation.

*Form function.* Given a reference cell $\{x_1, x_2, x_3, x_4\}$ labeled in such a way that for the lower-left corner $x_1$ we have $x_1 = (s,t)$, we define the *barycenter* *z* of the reference cell via $z(s,t) := (1/2 + s, 1/2 + t)$. Thus, $z({\mathbb {Z}}^2)= {{\mathbb {Z}}^2}^*$, where ${{\mathbb {Z}}^{2}}^*$ denotes the dual lattice of ${\mathbb {Z}}^2$. For an admissible configuration *y*, we define the *form function* on the dual lattice $\tau _y :{{\mathbb {Z}}^{2}}^* \rightarrow \{\diagdown , \diagup \}$ as the map assigning to each reference cell the form of the optimal cell in the deformed configuration. In other words, the deformation *y* maps a reference cell with barycenter *z*(*s*, *t*) to an optimal cell of form $\tau _y(z(s,t))$. In the sequel, we simply write $\tau$ for notational convenience.

**Fig. 3:**
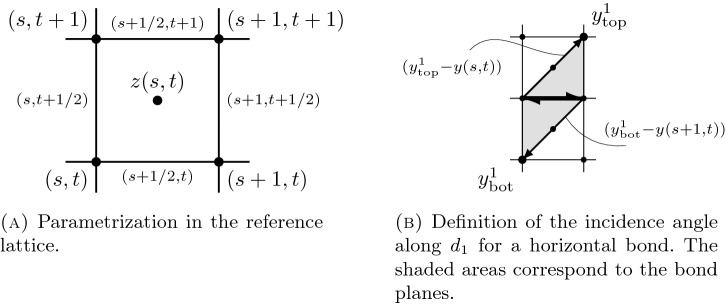
Notions for Theorem 2.2

*Incidence angles.* We define the diagonals $d_1 = (1,1)$ and $d_2 = (-1,1)$. For $i=1,2$, we indicate *signed incidence angles along the diagonal* $d_i$ for each bond of the configuration via the mappings $\gamma _i:(({\mathbb {Z}}+1/2) \times {\mathbb {Z}}) \cup ({\mathbb {Z}}\times ({\mathbb {Z}}+1/2)) \rightarrow [-\pi ,\pi ]$ defined as follows: first, for $s, t \in {\mathbb {Z}}$, $(s+1/2,t)$ parametrizes the horizontal bond in the reference lattice connecting (*s*, *t*) and $(s+1,t)$, and $(s,t+1/2)$ parametrizes the vertical bond in the reference lattice connecting (*s*, *t*) and $(s,t+1)$, see Fig. 3A. In the following, we explicitly give the definition of the incidence angle $\gamma _i(s+1/2,t)$, $i=1,2$, for horizontal bonds. The definition associated to vertical bonds follows analogously, up to a rotation of the reference lattice by $\pi /2$.

Consider a horizontal bond parametrized by $(s+1/2,t)$, which is shared by the two cells with barycenters $z(s,t-1) = (s+1/2,t-1/2)$ and $z(s,t) = (s+1/2, t+1/2)$. By $n^i_{\mathrm{top}}$ we denote the unit normal vector to the plane spanned by the points *y*(*s*, *t*), $y(s+1,t)$, and $y_{\mathrm{top}}^i := y((s,t)+v_i),$ with direction $(y(s+1,t) - y(s,t)) \times (y_{\mathrm{top}}^i - y(s,t))$, where for convenience we set $v_1 := d_1 = (1,1)$ and $v_2 := (0,1)$. Analogously, we let $n^i_{\mathrm{bot}}$ be the unit normal vector to the plane spanned by *y*(*s*, *t*), $y(s+1,t)$, and $y_{\mathrm{bot}}^i:= y((s+1,t)-v_i)$ with direction $(y(s,t) - y(s+1,t)) \times (y_{\mathrm{bot}}^i - y(s+1,t))$, see Fig. 3B.

Then, for all $s,t \in {\mathbb {Z}}$, the *signed incidence angles along the diagonal* $d_i$ of horizontal bonds are given by

2.15 $$\begin{aligned} \gamma _i(s+1/2, t) = \left\{ \begin{array}{rcl} \arccos (n^i_{\mathrm {top}}\cdot n^i_{\mathrm {bot}}) &{} \! \text {if} \! &{} (y^i_{\mathrm{top}}- y_{\mathrm{bot}}^i) \cdot (n^i_{\mathrm {top}} - n^i_{\mathrm{bot}}) \ge 0\\ -\arccos (n^i_{\mathrm {top}}\cdot n^i_{\mathrm {bot}}) &{} \! \text {if} \! &{} (y^i_{\mathrm{top}}- y_{\mathrm{bot}}^i) \cdot (n^i_{\mathrm {top}} - n^i_{\mathrm{bot}}) < 0. \end{array} \right. \qquad \end{aligned}$$

Making use of the introduced notation, we are now in the position of formulating our first result. This is a simplified version of the later Theorem 2.8 and provides *necessary* conditions on the existence of admissible configurations.

#### Theorem 2.2

(Basic structure of admissible configurations) There exists $\gamma ^* \in (0,\pi )$, depending only on $\theta$, such that for every admissible configuration $y:{\mathbb {Z}}^2\rightarrow {\mathbb {R}}^3$, possibly up to reorientation of the reference lattice, the following holds true:

- (Constant form function along $d_1$) We have $\tau (s,t) = \tau (s+1, t+1)$ for all $s,t \in {\mathbb {Z}}$.
- (Vanishing incidence angle along $d_1$) We have $\gamma _1(s+1/2,t) = 0 = \gamma _1(s,t+1/2)$ for all $s,t\in {\mathbb {Z}}$.
- (Incidence angle along $d_2$) It holds that $\gamma _2(s,t) = \gamma _2( s+1/2,t+1/2) \in \{ \pm \gamma ^*, 0\}$ for all $s,t\in \frac{1}{2}{\mathbb {Z}}$ with $s+t \in {\mathbb {Z}}+1/2$.

**Fig. 4:**
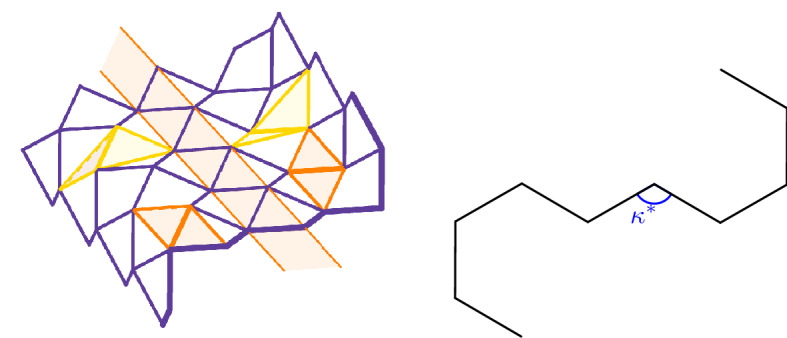
An admissible configuration (left). Since the form function is constant along the diagonal $d_1$, as indicated by the orange area, the same pattern repeats periodically and all necessary information is contained in one cross section as shown on the right. The angle $\kappa ^*$ is defined in (2.17). The defining bond planes for vertical (on the right) and horizontal (on the left) bonds of the incidence angles $\gamma _1$ (orange) and $\gamma _2$ (yellow) are marked, indicating that $\gamma _1= 0 \ne \gamma _2$

This theorem implies that ground states are essentially one-dimensional, in the sense that they can be characterized as two-dimensional deformations of one-dimensional chains, see Fig. 4. Indeed, due to $\tau$ being constant along $d_1$, any cross section along $d_2$ contains the same information. In particular, admissible configurations can be any combination of flat, rolled-up/down areas in relation to the fact that the incidence angle along $d_2$ can be 0 (flat areas), $-\gamma ^*$ (rolled-up areas) or $+\gamma ^*$ (rolled-down areas).

In the next subsections, we will present a refined version of Theorem 2.2, namely Theorem 2.8. We will show that Theorem 2.8 below implies Theorem 2.2. In Sect. 3 we then prove Theorem 2.8, which then also implies Theorem 2.2.

### Geometry of Optimal Cells and Construction of 4-Tiles

We aim at obtaining a complete characterization of admissible configurations, by resorting to so-called 4-*tiles*. To introduce this concept, we first need to investigate the geometry of optimal cells in more detail. First, we consider an admissible deformation *y* and an optimal cell of the configuration, consisting of the points $y_1,\ldots ,y_4$ and the corresponding midpoints $m_1 = (y_1 + y_3)/2$ and $m_2 = (y_2 + y_4)/2$, as indicated in Fig. 2. We denote the length of the diagonal by $2v:= \vert y_1 - y_3 \vert = \vert y_2 - y_4 \vert$. By the cosine rule we have

2.16 $$\begin{aligned} v = \sqrt{ (1-\cos \theta )/2 }. \end{aligned}$$

Setting $d := \vert y_1 - m_2 \vert = \vert y_3 - m_2 \vert = \vert y_2 - m_1 \vert = \vert y_4 - m_1 \vert$, we obtain by Pythagoras’ theorem $d = \sqrt{1-v^2}= \sqrt{(1+\cos \theta )/2}$. This allows us to calculate the *kink angle* $\kappa ^*$ of an optimal cell by

**Fig. 5:**
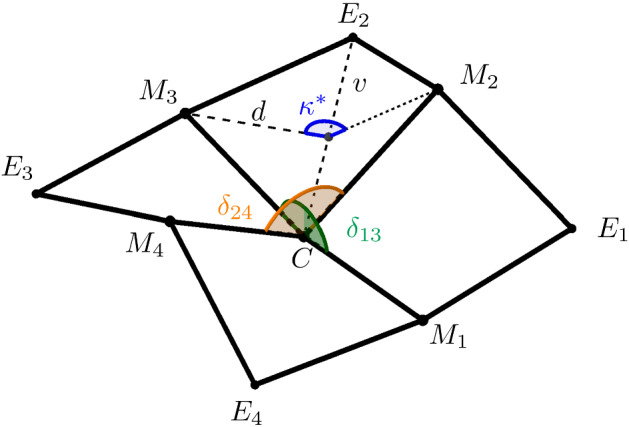
Example of a 4-tile with center *C*, middle points $M_1, \dots , M_4$ and corner points $E_1, \dots , E_4$. We have also indicated *v*, *d*, and $\kappa ^*$ of the optimal cell $\{C,M_2, E_2, M_3\}$

2.17 $$\begin{aligned} \kappa ^* = \pi - 2 \kappa , \quad \quad \text {where}\, \kappa :=\arccos (v/d) = \arctan (h/v), \end{aligned}$$

with $h = \sqrt{1-2v^2}$, see also Lemma 2.5. We refer to Fig. 5 with the optimal cell formed by $\lbrace C,M_2,E_2,M_3 \rbrace$ for an illustration. For $\theta = \pi /2$ we have $v/d = 1$, and thus $\kappa ^* = \pi$. In this case, as expected, optimal cells are flat. Let us firstly observe that an optimal cell is uniquely determined by the coordinates of three points and the choice of the cell form.

#### Lemma 2.3

(Optimal cell). Given any three points $y_1, y_2, y_4 \in {\mathbb {R}}^3$ of an optimal cell, i.e., points satisfying $\vert y_1 - y_4 \vert = \vert y_1 - y_2 \vert = 1$ and $\measuredangle y_4 y_1 y_2 = \theta$, there exists a unique fourth point $y^\diagdown _3$ and $y_3^\diagup$, respectively, such that $\{y_1, y_2, y_3^\diagdown , y_4\}$ is optimal of form $\diagdown$ and $\{y_1, y_2, y_3^\diagup , y_4\}$ is optimal of form $\diagup$.

For the proof, we refer to Sect. A.1. A priori, by prescribing only the common angle $\theta$, many configurations are conceivable as each optimal cell can be of form $\diagdown$ or form $\diagup$, and neighboring cells can in principle be attached to each other with an arbitrary incidence angle. Condition (2.14) is therefore essential to reduce the number of admissible deformations. To take (2.14) into account, we now consider sub-configurations consisting of four optimal cells which are arranged in a square sharing one common point. Such structures are called 4*-tiles*, and we refer to Fig. 5 for an illustration.

The point shared by all four optimal cells is called *center* and is denoted by *C*. The additional four points shared by two optimal cells are called *middle points* (as they are in the middle of the boundary of the 4-tile), are denoted by $M_i$ for $i = 1,\dots ,4$, and are labeled counter-clockwise such that

$$\begin{aligned} y^{-1}(M_1) - y^{-1}(M_3) = 2e_1\quad \text {and} \quad y^{-1}(M_2) - y^{-1}(M_4) = 2e_2. \end{aligned}$$

By construction, we have $\measuredangle M_i \, C \, M_{i+1} = \theta < \pi /2$ which implies that the five points *C* and $(M_i)_{i=1}^4$ cannot be coplanar. We introduce the *nonplanarity angles* $\delta _{13}$ and $\delta _{24}$ by

2.18 $$\begin{aligned} \delta _{13} := \measuredangle M_1 \, C \, M_3 \quad \text { and } \quad \delta _{24}:= \measuredangle M_2 \, C \, M_4. \end{aligned}$$

Note that $|\delta _{13}- \pi |$ and $|\delta _{24}- \pi |$ indicate how far the five points *C* and $(M_i)_{i=1}^4$ are from being coplanar, and again refer to Fig. 5 for an illustration. The nonplanarity angles $\delta _{13}$ and $\delta _{24}$ are related by the following lemma.

#### Lemma 2.4

(Nonplanarity angles). The nonplanarity angles $\delta _{13}$ and $\delta _{24}$ satisfy

2.19 $$\begin{aligned} \cos \big ( \delta _{13} / 2\big ) \cos \big ( \delta _{24}/2\big ) = \cos \theta . \end{aligned}$$

In particular, $\delta _{13}$ and $\delta _{24}$ coincide if and only if

$$\begin{aligned} \delta _{13} = \delta _{24} = \delta _\theta = 2 \arccos (\sqrt{\cos \theta }). \end{aligned}$$

Indeed, by (2.14) we always have $\delta _{13} = \delta _{24}$ for every 4-tile of an admissible configurations since $M_1,\, C,\, M_3$ and $M_2, \, C,\, M_4$ fulfill the condition in (2.14). This yields that $\delta _\theta = 2\arccos (\sqrt{\cos \theta })$ is solely determined by $\theta$. The proof relies on the geometry of optimal cells, i.e., on assumptions (2.12) and (2.13), and will be given in Sect. A.1.

We denote the four corner points of the 4-tile by $E_i$, $i = 1,\dots ,4$, as indicated in Fig. 5. For the classification of all different 4-tiles, it is convenient to frame 4-tiles in a *reference position*, as given in the following proposition.

#### Lemma 2.5

(Reference position). (i) By applying a suitable isometry, every 4-tile can be positioned in such a way that the center *C* coincides with the origin, and we have

$$\begin{aligned} M_1 = (s,0,\varsigma h), \quad M_2 = (0,s,\varsigma h), \quad M_3 = (-s,0,\varsigma h), \quad M_4 = (0,-s,\varsigma h), \end{aligned}$$

where $s = \sqrt{2}v$ (see (2.16)), $h = \sqrt{1-2v^2}$, and $\varsigma \in \lbrace -1,1 \rbrace$.

(ii) Fixing $\varsigma \in \lbrace -1,1 \rbrace$, and the form of each of the four optimal cells, the positions of $(M_i)_{i=1}^4$ and $(E_i)_{i=1}^4$ are uniquely determined, up to isometry.

For the proof, we again refer to Sect. A.1. Lemma 2.5 entails that the middle points $(M_i)_{i = 1}^4$ are coplanar. For this reason, we call 4-tiles *coplanar* in the following. By (2.14) coplanarity is a necessary condition for the admissibility of 4-tiles.

**Fig. 6:**
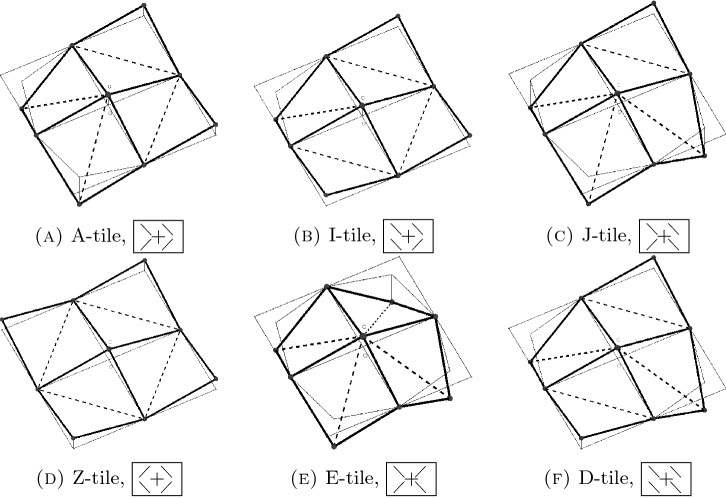
Representative 4-tiles of each class

In view of Lemma 2.5(ii), there are 32 different *types* of 4-tiles. Indeed, there are $2^4 = 16$ possibilities to distribute either a form $\diagdown$ or a form $\diagup$ optimal cell to the four positions of a 4-tile. Additionally, one can do this construction for $\varsigma =1$ or $\varsigma =-1$. As we show next, the different types can be classified into six *classes* which are invariant under rotation by $\pi /2$ and reflection along the $e_1$-$e_2$-plane, see Table 1. A representative of each class is shown in Fig. 6. The names of the classes are inspired by their geometry: the I-tile is intermediate between the zigzag-shaped Z-tile and the diagonally rolled-up D-tile (cf. the example in (2.22)). Similarly, the J-tile joins the arrowhead-shaped A-tile with the E-tile, whose periodic pattern resembles to egg cartons.

To denote a 4-tile we use a matrix-like notation, where the form of the optimal cell in the square is represented by $\diagdown$ or $\diagup$ in the respective position in the matrix. The case of $\varsigma =-1$ is indicated with a $+$-symbol in the center of the matrix, and $\varsigma =1$ is denoted with a −-symbol. We use this notation since, given a 4-tile in reference position, we have that for $i=1,\ldots ,4$ the center satisfies $(C - M_i) \cdot e_3 > 0$ if $\varsigma =-1$ (e.g. in Fig. 6D) and $(C - M_i) \cdot e_3 < 0$ if $\varsigma =1$ (e.g. in Fig. 5), see Lemma 2.5(i).

**Table 1:** Full classification of all possible 4-tiles

A-tile	, , , , , , ,	Figure 6A
I-tile	, , , , , , ,	Figure 6B
J-tile	, , , , , , ,	Figure 6C
Z-tile	,	Figure 6D
E-tile	,	Figure 6E
D-tile	, , ,	Figure 6F

Reflection of a 4-tile in reference position with respect to the $e_1$-$e_2$-plane interchanges the index $+$ with −. Moreover, $\diagup$ and $\diagdown$ are exchanged, as observed in Sect. 2.2. Also a rotation by $\pi /2$ interchanges the forms of the optimal cells, i.e., swaps $\diagdown$ and $\diagup$, see again Sect. 2.2. In addition, note that, by applying a $\pi /2$ rotation, one needs to permute the entries of the matrix accordingly, e.g.,

$$\begin{aligned} A \mapsto A^T \left( \begin{array}{cc} 0 &{}\quad 1 \\ 1 &{}\quad 0 \end{array} \right) \end{aligned}$$

for a clockwise rotation of the entries.

As an example, rotation leaves the 4-tile  invariant, as interchanging $\diagup$ and $\diagdown$ yields  and the rotation of the entries then leads to . However, rotating  clockwise, i.e., first swapping $\diagdown$ and $\diagup$ to obtain  and then rotating the entries to , yields an 4-tile of the same class, but with different type, see Table 1.

### Boundary Orientation and Boundary Angles

In this subsection, we further refine the characterization of 4-tiles by introducing a notion of boundary orientation. To this end, consider a 4-tile with notation as indicated in Fig. 5, placed in reference position. We call three points $E_{i-1}$, $M_i,$ and $E_i$, and the two bonds in between a *boundary* of the 4-tile, where the indices have again to be understood modulo 4. We define the *boundary orientation* of $E_{i-1} \, M_i \, E_i$ by

2.20 $$\begin{aligned} {\mathcal {O}}(E_{i-1} \, M_i \, E_i) := \left\{ \begin{array}{ccc} \wedge &{}\quad \text {if} &{} (E_i +E_{i-1})\cdot e_3 /2 < M_i \cdot e_3 , \\ \vee &{}\quad \text {if} &{} (E_i +E_{i-1})\cdot e_3 /2 > M_i \cdot e_3 , \end{array} \right. \end{aligned}$$

and the corresponding *boundary angle* by

2.21 $$\begin{aligned} \measuredangle E_i \, M_i \, E_{i-1}. \end{aligned}$$

Intuitively, the orientation describes the fact that the boundary points upwards (orientation $\wedge$) or downwards (orientation $\vee$), see Fig. 6 for an illustration. Boundary orientation and boundary angle are crucial for classifying admissible configurations as they provide compatibility conditions for neighboring 4-tiles. To formalize this, we now introduce the notion of *attached 4-tiles*.

Given two 4-tiles *T* and ${{\tilde{T}}}$ with centers *C* and ${{\tilde{C}}}$, we say that the 4-tiles are *attached to each other* if $y^{-1}(C) - y^{-1}({{\tilde{C}}}) \in \lbrace 2 e_1, - 2 e_1, 2 e_2 , -2 e_2 \rbrace$. Note that *T* and ${{\tilde{T}}}$ share exactly one of the middle points $(M_i)_{i=1}^4$ and $({{\tilde{M}}}_i)_{i=1}^4$ (and the adjacent two corner points). This shared middle point is the center of the so-called *middle 4-tile* which is formed by two optimal cells of *T* and two optimal cells of ${{\tilde{T}}}$.

The following result will be a key tool for the classification of admissible configurations.

#### Lemma 2.6

(Attachment of two 4-tiles) If two 4-tiles are attached to each other, the boundary angles and the boundary orientation at the shared boundary coincide. If the boundary orientation is $\wedge$, the corresponding middle 4-tile satisfies $\varsigma = -1$ (see Lemma 2.5(i)), otherwise we have $\varsigma = 1$.

Lemma 2.6 will be proved in Sect. A.2. The statement delivers necessary conditions for attaching two 4-tiles. In fact, a crucial idea for proving the main theorem, Theorem 2.8, is excluding many situations by checking that boundary angles or boundary orientations do not match. In particular, this reasoning will allow us to prove that admissible configurations *exclusively* contain Z-, D-, and I-tiles. To ease the readability, from now on we include the boundary orientation in the notation, at least for the relevant tiles, i.e., the Z-, D-, and I-tiles. This allows for an easy check whether the boundary orientations match or not.

On lateral boundaries, we denote boundaries with orientation $\wedge$ by <. Likewise, lateral boundaries with boundary orientation $\vee$ are indicated by >. Table 2 gives an overview of admissible 4-tiles with the new notation.

**Table 2:** A table of all admissible Z-, D-, and I-tiles with corresponding boundary orientations

Z-tile	,
D-tile	, , ,
I-tile	, , , , , , ,

In the notation, we also denote corner points pointing downwards with $\circ$ and corner points pointing upwards with $\bullet$ (of course, always assuming that the 4-tile is in reference position). As an example, we refer to (b) and (f) in Fig. 6 for  and , respectively. Note that this notation is not part of the characterization of types, but is included only to visualize the directions along which the boundary rolls up or down, respectively. (In fact, a $+$ in the center along with $\diagup$ pointing towards $+$ yields $\circ$ in the corresponding corner. In a similar fashion, a − in the center along with $\diagup$ *not* pointing towards − yields $\bullet$.) This notation facilitates to determine the class of the 4-tile as Z-tiles have no $\bullet$/$\circ$, D-tiles have two, and I-tiles have exactly one.

#### Lemma 2.7

(Boundary orientations). The boundary orientations of the different boundaries of the Z-, D-, and I-tiles are given as indicated in Table 2.

Lemma 2.7 will be proved in Sect. A.2. We close this subsection with an example illustrating Lemma 2.6. Let us attach the Z-tile  and the D-tile . From the notation we can directly see that by attaching via2.22the boundary orientation match at the shared boundary, i.e., the 4-tiles can be attached to each other provided that also the boundary angles coincide. (This indeed holds true, as we will see later in Lemma 3.1.) The type of the middle 4-tile can be determined directly by considering the forms of the four optimal cells in the middle, i.e., . As the shared boundary has orientation >, which corresponds to $\vee$, Lemma 2.6 implies that the middle 4-tile satisfies $\varsigma = 1$. The latter implies a −-symbol in the middle of the matrix, see the discussion below Lemma 2.5. Therefore, the middle 4-tile is the I-tile . Clearly, the procedure applies to all combinations of 4-tiles.

### Main Result: Characterization in Terms of 4-Tiles

After having introduced the necessary notation and concepts in the previous subsections, we are ready to formulate our main result on the characterization of admissible configurations in terms of 4-tiles. To this end, we need a variant of the form function, the so-called *type functions*: consider an admissible deformation *y* and let $S_1 = 0$, $S_2 = (1,0)$, $S_3=(0,1)$, and $S_4 = (1,1)$. For $i=1,\ldots ,4$, we let $\sigma _i$ be the function defined on $2{\mathbb {Z}}^2$ such that $\sigma _i(k,l)$ for $(k,l) \in 2{\mathbb {Z}}^2$ indicates the type of the 4-cell with center $y(S_i + (k,l))$. The four different functions account for the fact that a translation of ${\mathbb {Z}}^2$ by (0, 0), (1, 0), (0, 1), or (1, 1) leaves the deformed configuration invariant, but groups together different optimal cells to form 4-tiles.With this definition at hand, we now state the main result of this paper.

#### Theorem 2.8

(Characterization of all admissible configurations) A deformation *y* is admissible if and only if, possibly up to rotation of the lattice ${\mathbb {Z}}^2$ by $\pi /2$, the following holds true:

Only particular types of Z-, D-, and I-tiles are admissible, namely, for $i=1,\ldots ,4$ we have2.23Moreover, the type function is constant along $d_1$, i.e., $\sigma _i(s,t) = \sigma _i(s+2, t+2)$ for all $s,t \in 2{\mathbb {Z}}$ and the following *matching conditions* are satisfied:

1. for all $s,t \in 2{\mathbb {Z}}$ we have
2. for all $s,t \in 2{\mathbb {Z}}$ we have

The theorem gives a *complete characterization* of all admissible configurations. First, it shows that only Z-, D-, and I-tiles are admissible. More precisely, we see that only such D-, and I-tiles from Table 2 are admissible, which roll-up/down along the same diagonal, and that the type function is constant along the other diagonal. In particular, no change between the direction of rolling-up/down is admissible. This observation allows for a clear geometric interpretation: Z-tiles correspond to flat areas and D-tiles induce rolled-up/down areas. In order to match such 4-tiles, the I-tile arises naturally as a combination of the Z-tile and D-tile. (See, e.g., Fig. 6B, which is a D-tile left and a Z-tile right. See also the example in (2.22).) Clearly, rolling-up/down exclusively along the other diagonal is admissible as well, corresponding exactly to the other collection of D-, and I-tiles from Table 2. However, after a rotation of the lattice ${\mathbb {Z}}^2$ by $\pi /2$, one can always reduce to (2.23). Eventually, the matching conditions (M1) and (M2) further restrict the admissible combination of 4-tiles, and account for the fact that the boundary orientations at shared boundaries of two attached 4-tiles need to match, see Lemma 2.6. We close this discussion by noting that the characterization cannot be simplified further, i.e., there are indeed admissible configurations *y* which contain all eight types given in (2.23).

Let us now stress that Theorem 2.8 implies Theorem 2.2. To see this, we observe that the type functions $\sigma _i$, $i=1,\ldots ,4$, are constant along the diagonal $d_1$. This along with the fact that all types in (2.23) have the same form of optimal cell ($\diagdown$ or $\diagup$) along the diagonal $d_1$ (i.e., in the lower left and upper right entry) shows that the form function $\tau$ introduced in Sect. 2.2 satisfies $\tau (s,t) = \tau (s+1, t+1)$ for all $s,t \in {\mathbb {Z}}$.

The fact that all incidence angles along $d_1$ vanish and that all incidence angles along $d_2$ lie in $\lbrace 0,\gamma ^*,-\gamma ^*\rbrace$ (with the property that the value is constant along $d_1$) follows by an elementary computation. We defer the exact calculation to Appendix A.4. At this stage, we only mention that inside Z-tiles, all incidence angles along both diagonals are equal to zero. On the other hand, for the D-tile  the incidence angle along $d_2$ is $\gamma ^*$ and for  it is $-\gamma ^*$. I-tiles have incidence angles 0 and $\pm \gamma ^*$, where the sign depends on $\bullet$ or $\circ$ in the notation.

## The Proof of the Main Theorem

This section is devoted to the proof of Theorem 2.8. This hinges on two facts, namely, that (1) attaching two 4-tiles is only possible if the boundary orientation at shared boundaries match and (2) that such attachment needs to lead to an admissible, i.e., coplanar middle 4-tile. Firstly, we use these ideas to show that actually only Z-, D-, and I-tiles are admissible, see Proposition 3.2. In a second step, we further restrict the set of admissible types by showing that D- and I-tiles necessarily need to roll-up/down along the same diagonal, see Proposition 3.3. This is achieved by considering four 4-tiles arranged in a square and exploiting the aforementioned compatibility conditions. With similar techniques, we subsequently show that along one diagonal the type has to be constant, see Proposition 3.4. Eventually, we provide another auxiliary result (Proposition 3.5) stating that four 4-tiles arranged in a square can be indeed realized by an admissible configuration *y* if all compatibility conditions, including the matching conditions stated in Theorem 2.8, are satisfied. With these results at hand, we are then able to prove Theorem 2.8.

### Admissible Classes of 4-Tiles

In this subsection, we show that admissible configurations contain only Z-, D-, and I-tiles and that pairs of such tiles can be attached. This is achieved in two steps. We start by calculating the different boundary angles introduced in (2.21). Then, by discussing the possibility of attaching two 4-tiles along a boundary with the same boundary angle and the same boundary orientation, see (2.20), we are able to show that Z-, D-, and I-tiles are admissible, while E-, A-, and J-tiles are not.

We start by observing that there are exactly three different boundary types. In view of Lemma 2.5, we see that the three points forming a boundary (e.g., $E_{i-1}$, $M_i$, and $E_i$, see Fig. 5) are completely characterized by $\varsigma \in \lbrace -1,1\rbrace$ and the form, i.e., form $\diagdown$ or form $\diagup$, of the two optimal cells adjacent to the boundary. (Strictly speaking, in Lemma 2.5(ii), this was only shown once the forms of all four optimal cells are fixed, but the argument clearly localizes at each boundary.)

This leads to at most $2^3 = 8$ different boundary types, as indicated in Table 3. Given a 4-tile in reference position, the boundary type remains invariant under reflection of the 4-tile along the $e_1$-$e_2$-plane and the $e_2$-$e_3$-plane. This shows that the number of different boundary types reduces to three. We indicate the corresponding boundaries as *Z-, D-, and E-boundaries*, respectively, as the corresponding 4-tiles have exclusively such boundaries, compare also Table 3 with Table 1. We also mention that I-tiles have both Z- and D-boundaries, but no E-boundaries, and that J- and A-tiles contain E-boundaries.

**Table 3:** Classification of the three types of boundaries

Z-boundary	D-boundary	E-boundary
,	, ,,	,

#### Lemma 3.1

(Boundary angles) The Z-boundary angle and D-boundary angle of coplanar 4-tiles are given by $\delta _\theta = 2\arccos \left( \sqrt{\cos \theta }\right)$. The E-boundary angle of coplanar 4-tiles is strictly smaller than $\delta _\theta$.

#### Proof

We start by considering the Z-boundary angle. Without restriction we consider a Z-tile in reference position with notation as indicated in Fig. 5, satisfying $M_2 = (0,s,h)$ for $s,h>0$, where *s* and *h* are given in Lemma 2.5. We observe that the isometry $x = (x_1,x_2,x_3) \mapsto (x_1,x_2,-x_3) + (0,s,h)$ maps $M_1$ to $E_1$, *C* to $M_2$, and $M_3$ to $E_2$. This yields that the Z-boundary angle coincides with $\delta _\theta$, see (2.14) and (2.18). The fact that the D-boundary angle coincides with the Z-boundary angle is postponed to Corollary A.3, and relies on the fact that two 4-tiles with the respective boundaries can be attached to each other, cf. Lemma A.2.

Eventually, we show that the E-boundary angle is strictly smaller. To this end, we let $$E_1= (s,s,0)$$, $$M_2= (0,s,h) $$, $$E_2 = (-s,s,0) $$ be again the points of the Z-tile considered above. The corresponding points of an E-tile in reference position are denoted by $${{\tilde{E}}}_1$$, $${{\tilde{M}}}_2$$, and $${{\tilde{E}}}_2$$. (They are obtained by changing the form of the optimal cells containing $$E_1$$ and $$E_2$$, respectively.) By simple geometric considerations we find

3.1 $$\begin{aligned} {{\tilde{E}}}_1 = E_1 + (-p,-p,q), \quad \quad {{\tilde{M}}}_2 = M_2, \quad \quad {{\tilde{E}}}_2 = E_2 + (p,-p,q) \end{aligned}$$

for some $p,q>0$. One can check that $q = {{\tilde{E}}}_1 \cdot e_3 = {{\tilde{E}}}_2 \cdot e_3 > 2h$, see Lemma A.1(iv) below. Given that $|{{\tilde{E}}}_1 - {{\tilde{M}}}_2| = | {{\tilde{E}}}_2 - {{\tilde{M}}}_2| = 1$, the E-boundary angle is calculated by $\arccos (({{\tilde{E}}}_1 - {{\tilde{M}}}_2) \cdot ({{\tilde{E}}}_2 - {{\tilde{M}}}_2))$. We now compute by using (3.1) and $q > 2h$ that

$$\begin{aligned} ({{\tilde{E}}}_1 - {{\tilde{M}}}_2) \cdot ({{\tilde{E}}}_2 - {{\tilde{M}}}_2)= & {} ({E}_1 - {M}_2) \cdot ({E}_2 - {M}_2) + \begin{pmatrix} -p \\ -p \\ q \end{pmatrix} \cdot \begin{pmatrix} -s \\ 0 \\ -h \end{pmatrix} \\&+ \begin{pmatrix} p \\ -p \\ q \end{pmatrix} \cdot \begin{pmatrix} s \\ 0 \\ -h \end{pmatrix} + \begin{pmatrix} -p \\ -p \\ q \end{pmatrix} \cdot \begin{pmatrix} p \\ -p \\ q \end{pmatrix} \\= & {} ({E}_1 - {M}_2) \cdot ({E}_2 - {M}_2) + 2ps - 2qh + q^2 \\> & {} ({E}_1 - {M}_2) \cdot ({E}_2 - {M}_2). \end{aligned}$$

As $\delta _\theta = \arccos (({E}_1 - {M}_2) \cdot ( {E}_2 - {M}_2))$ and $\arccos$ is strictly decreasing on $[-1,1]$ we find that the E-boundary angle is smaller than $\delta _\theta$. This concludes the proof. $\square$

#### Proposition 3.2

(Nonadmissible classes of 4-tiles). An admissible configuration does not contain E-, A-, and J-tiles.

#### Proof

Suppose by contradiction that the configuration contains a 4-tile of class E, A, or J. As each E-, A-, or J-tile contains at least one E-boundary, see Tables 1 and 3, by Lemma 2.6 and Lemma 3.1 we deduce that the configuration contains at least two adjacent 4-tiles in these three classes such that the shared boundary has an E-boundary angle. For the corresponding middle 4-tile between the two 4-tiles we thus get that the corresponding $\delta _{13}$ or $\delta _{24}$ as defined in (2.18) coincides with the E-boundary angle which is strictly smaller than $\delta _\theta$ by Lemma 3.1. On the other hand, by (2.14) we have $\delta _{13} = \delta _{24} =\delta _\theta$ for the nonplanarity angles of the middle tile, a contradiction. $\square$

### Proof of the Main Result

In this subsection we give the proof of Theorem 2.8. The argument rests upon two propositions, showing that only certain arrangements of Z-, D-, and I-tiles are admissible. A third auxiliary result verifies that such arrangements are indeed admissible. We start by stating these results, whose proofs are postponed to the next subsections. Recall the notation of the 4-tiles in Table 2.

#### Proposition 3.3

(Roll-up/down along one diagonal). Consider any four adjacent 4-tiles of class Z, D, or I of an admissible configuration arranged in a square. Then all D- and I-tiles locally roll-up/down along the same diagonal, i.e., the type of the four 4-tiles is either exclusively contained in ${\mathcal {A}}$ or exclusively contained in ${\mathcal {B}}$, where3.2and3.3

Note that ${\mathcal {B}}$ can be obtained from ${\mathcal {A}}$ through a rotation of the reference lattice by $\pi /2$, and vice versa. The proposition shows that locally only 4-tiles which roll along the same diagonal can be attached to each other. The following result states that locally admissible configurations have the same type along one of the diagonals.

#### Proposition 3.4

(Arrangements along diagonals). Consider four adjacent 4-tiles of an admissible configuration with types either in ${\mathcal {A}}$ or in ${\mathcal {B}}$, see (3.2)–(3.3), arranged in a square and denoted byIf the types are in ${\mathcal {A}}$, we have ${\mathfrak {B}} = {\mathfrak {D}}$, and if the types are in ${\mathcal {B}}$, we have ${\mathfrak {A}} = {\mathfrak {C}}$.

The previous two results yield restrictions for the arrangement of 4-tiles in admissible configurations. The next result shows that such arrangements are indeed admissible.

#### Proposition 3.5

(Admissible arrangements of 4-tiles).

(i) If two coplanar 4-tiles in ${\mathcal {A}}$ are attached along a boundary with matching boundary orientation, the resulting middle 4-tile is a coplanar 4-tile in ${\mathcal {A}}$.

(ii) If four adjacent coplanar 4-tiles with types in ${\mathcal {A}}$ are arranged as3.4such that ${\mathfrak {B}} = {\mathfrak {D}}$ and such that the four 4-tiles satisfy the matching conditions (M1)–(M2) stated in Theorem 2.8, there exists an admissible deformation $y:\lbrace 0,1,2,3,4\rbrace ^2 \rightarrow {\mathbb {R}}^3$ such that the 4-tiles of $y(\lbrace 0,1,2,3,4\rbrace ^2 )$ have the types indicated in (3.4).

A similar statement holds for 4-tiles with types in ${\mathcal {B}}$ by rotation of the reference lattice by $\pi /2$. We are now in a position to prove our main result.

#### Proof of Theorem 2.8

*Step 1:* $\Rightarrow$. We recall the definition of $\sigma _i$, $i=1,\ldots ,4$, before the statement of Theorem 2.8. Without restriction we only consider $\sigma _1$ in the following proof. By Proposition 3.2 we have that the configuration only contains Z-, D-, and I-tiles.

We next show that all types are either in ${\mathcal {A}}$ or in ${\mathcal {B}}$, see (3.2)–(3.3), i.e., rolling up/down occurs at most along one diagonal. Assume by contradiction that there were two 4-tiles rolling along different diagonals, i.e., $T_1 \in {\mathcal {A}} \setminus {\mathcal {B}}$ and $T_2 \in {\mathcal {B}} \setminus {\mathcal {A}}$. Choose $s_i,t_i \in 2{\mathbb {Z}}$, $i=1,2$, such that $\sigma _1(s_1,t_1) = T_1$ and $\sigma _1(s_2,t_2) = T_2$. By Proposition 3.3 we can apply Proposition 3.4 and thus find $\sigma _1(s_1+r,t_1+r) = T_1$ and $\sigma _1(s_2+r',t_2-r') = T_2$ for all $r,r' \in 2{\mathbb {Z}}$. For a particular choice of *r* and $r'$ this entails $T_1 = T_2$ or that $T_1$ is adjacent to $T_2$. In both cases, we obtain a contradiction to Proposition 3.3.

This shows that all types of 4-tiles are either in ${\mathcal {A}}$ or ${\mathcal {B}}$. Up to a rotation of the reference lattice by $\pi /2$, we may suppose that all types of 4-tiles lie in ${\mathcal {A}}$, which corresponds to the notation of Theorem 2.8. By Proposition 3.4 we get that the type function is constant along $d_1$, i.e., $\sigma _i(s,t) = \sigma _i(s+2, t+2)$ for all $s,t \in 2{\mathbb {Z}}$ and all $i=1,\ldots ,4$.

It remains to show that the *matching conditions* (M1) and (M2) hold true as indicated in the statement. These properties rely on the fact that the boundary orientations of each two attaching 4-tiles need to match, cf. Lemma 2.6.

We only prove matching condition (M1) as the proof for (M2) follows along similar lines. Since the type function is constant along $d_1$, i.e., $\sigma _1(s,t) = \sigma _1(s+2, t+2)$ ($s,t \in 2{\mathbb {Z}}$), for any $s,t \in 2{\mathbb {Z}}$ such that , we have one of the two possibilitieswhere the boundaries of the 4-tiles with type $\sigma _1(s,t) = \sigma _1(s+2,t+2)$ are depicted with solid lines. The given boundary orientations and Lemma 2.6 imply that only a 4-tile from (compare Table 2)  can be attached in the blank position top left indicated by the dotted 4-tile (where its straight boundaries represent arbitrary boundary orientations). Within the class of admissible 4-tiles ${\mathcal {A}}$ in (3.2), exactly the four choices  match this boundary orientation. Conversely, for $s,t \in 2{\mathbb {Z}}$ such that  an arrangement as above yields one of the two possibilitiesHowever, due to the given boundary orientations, the 4-tiles in

 are the only 4-tiles from ${\mathcal {A}}$ which can be attached in the blank position bottom-right, again indicated with the dotted 4-tile. This concludes the check of the matching conditions (M1).

*Step 2:* $\Leftarrow$. The existence of an admissible configuration $y:{\mathbb {Z}}^2 \rightarrow {\mathbb {R}}^3$ follows directly from Proposition 3.5(ii) and an induction argument. Indeed, (2.12) and (2.13) are satisfied since each cell is optimal. To see (2.14), it suffices to check that all 4-tiles are coplanar. In fact, then (2.14) follows from Lemma 2.4. First, by construction in Proposition 3.5(ii) we get that all 4-tiles related to the type function $\sigma _1$ are coplanar. By using Proposition 3.5(i) we find that also the 4-tiles related to the other type functions $\sigma _i$, $i=2,3,4$, are in ${\mathcal {A}}$ and are coplanar. This shows that all 4-tiles are coplanar, as desired. $\square$

### Rolling Along One Diagonal

This subsection is devoted to the proof of Proposition 3.3. The proof fundamentally relies on Lemma 2.6, i.e., the fact that the boundary orientations of attached 4-tiles match. To this end, we will make extensive use of the matrix diagrams introduced in Table 2 in order to exclude certain arrangements of 4-tiles. Unfortunately, not all nonadmissible cases can be ruled out by such compatibility analysis and we also need to consider some more refined tools, based on the real three-dimensional geometry of the 4-tiles. For this reason, we will use the following lemma concerning the attachment of four coplanar 4-tiles. Recall the types of 4-tiles ${\mathcal {A}}$ and ${\mathcal {B}}$ introduced in (3.2)–(3.3), as well as the different types of boundaries in Table 3.

#### Lemma 3.6

(Arrangements of four 4-tiles) Consider four adjacent 4-tiles of an admissible configuration with types either in ${\mathcal {A}}$ or in ${\mathcal {B}}$, see (3.2)–(3.3), arranged in a square and denoted by3.5Then: (i) If three tiles are Z-tiles and one tile is an D-tile, then the D-tile is in $\lbrace {\mathfrak {A}}, {\mathfrak {C}}\rbrace$ (case ${\mathcal {A}}$) or in $\lbrace {\mathfrak {B}}, {\mathfrak {D}}\rbrace$ (case ${\mathcal {B}}$).

(ii) If two tiles are Z-tiles and two tiles are D-tiles, then the Z-tiles are arranged along one diagonal and the D-tiles along the other diagonal.

(iii) If three tiles are D-tiles and one tile is a Z-tile, then the Z-tile is in $\lbrace {\mathfrak {A}}, {\mathfrak {C}}\rbrace$ (case ${\mathcal {A}}$) or in $\lbrace {\mathfrak {B}}, {\mathfrak {D}}\rbrace$ (case ${\mathcal {B}}$).

(iv) The arrangement3.6is not admissible.

We postpone the proof of this lemma to Appendix A.3 and proceed with the proof of Proposition 3.3.

#### Proof of Proposition 3.3

We proceed in two steps: in Step 1 we show that two attached 4-tiles cannot roll-up/down along different diagonals. In Step 2 we show that in four adjacent 4-tiles arranged in a square, the two pairs of diagonal 4-tiles cannot roll-up/down along different diagonals. These two steps imply the statement.

*Step 1: Attached* 4*-tiles.* Up to interchanging the roles of $\bullet$ and $\circ$, and up to reflection along the $e_1$- or the $e_2$-axis, there are six different cases to address:Here, the symbol  is a placeholder both for the corresponding I-tile  and the D-tile  . The meaning of the other symbols is analogous. For the proof, we refer the reader to Table 2 which collects all possible 4-tiles.

*Case 1:* . This case leads to a contradiction to Proposition 3.2 as necessarily the middle 4-tile is the A-tile . As an example, among the four possibilities, we consider the case where both 4-tiles are I-tiles. In this case, we have .

*Case 2:* . This case ensues if two 4-tiles with different boundary orientations are attached, which contradicts Lemma 2.6. As an example, among the four possibilities, we consider the case where both 4-tiles are D-tiles. In this case, we have .

*Case 3:* . First, if both 4-tiles are D-tiles, then up to a reflection along the $e_2$-axis, we are in Case 1 and obtain a contradiction as explained before. In the case that one is a D-tile and the other is an I-tile, we obtain a contradiction to Lemma 2.6 as then the boundary orientations do not match. In fact, these two last cases are  and .

We can therefore assume that both 4-tiles are I-tiles, i.e., take the form . We will now consider which 4-tiles are admissible on top of the given 4-tiles. Since we have already ruled out Case 1 and the boundary orientations need to match by Lemma 2.6, we see that on top of the left I-tile we can only have , , , , or , and on top of the right I-tile we can only have , , , , or . In any case, the 4-tile in the middle of the four considered 4-tiles, will be an A-tile of the form  or . This contradicts Proposition 3.2 and concludes the proof of Case 3.

*Case 4:* . If both 4-tiles are D-tiles, then up to a reflection along the $e_2$-axis, we are in Case 2 and obtain a contradiction as explained before. If both 4-tiles are I-tiles, we have , i.e., the boundary orientations are different and we obtain a contradiction to Lemma 2.6. The two remaining possibilities are  and . We prove the contradiction only for the first configuration as the second configuration can be treated along similar lines. In order to do so, we proceed as in Case 3 and attach 4-tiles at the top, yielding3.7In (3.7), the straight dotted lines encompass all possible boundary orientations. We start by noting that the I-tile in the middle of  is of the form .

Since we have already ruled out Case 1 and the boundary orientations need to match by Lemma 2.6, only the 4-tiles  can be attached on top of the D-tile (left), i.e., at position ${\mathfrak {A}}$. Similarly, on top of the I-tile (right) at position ${\mathfrak {B}}$ we can only attach the 4-tiles , see Table 2. As the boundary orientations between ${\mathfrak {A}}$ and ${\mathfrak {B}}$ have to match as well, there are only eight possibilities of the upper two 4-tiles which are indicated in the first two columns of Table 4. The two upper 4-tiles ${\mathfrak {A}}$ and ${\mathfrak {B}}$ form middle 4-tiles which are indicated in the third column of Table 4. Note that the 4-tile attached on the bottom of this 4-tile is exactly the middle 4-tile between the original two 4-tiles, i.e., . Therefore, in the first four cases we obtain a contradiction to Lemma 2.6 since the boundary orientations of the shared boundary of the two middle 4-tiles do not match.

For the second four cases we need a different argument instead. To this end, we consider also the middle 4-tile between the D-tile and ${\mathfrak {A}}$ (left middle 4-tile) and the middle 4-tile between the I-tile and ${\mathfrak {B}}$ (right middle 4-tile), see the last two columns in Table 4. We observe that in none of the cases the boundary orientations of the shared boundary of the left and right middle 4-tiles match. This is again a contradiction to Lemma 2.6, concluding the check of Case 4.

**Table 4:** The eight different cases considered in Case 4

At ${\mathfrak {A}}$	At ${\mathfrak {B}}$	Middle 4-tile ${\mathfrak {A}}$–${\mathfrak {B}}$	Middle 4-tile left	Middle 4-tile right
				
				
				
				
				
				
				
				

*Case 5:*  or : Without restriction we address only the first case as the second can be treated analogously (and, in fact, obtained by a rotation). We have to distinguish two cases. Firstly, the 4-tile on the left is a D-tile, i.e., . Then up to a reflection along the $e_2$-axis, we are in Case 1 and obtain a contradiction as explained before. Secondly, if the left 4-tile is not a D-tile, it has to be an I-tile. We obtain the two possible configurations  and  which both contradict Lemma 2.6 as the boundary orientations do not match.

*Case 6:*  or . Without restriction we address only the first case as the second can be treated analogously. If the 4-tile on the left is a D-tile, then we have the two possibilities  and . Thus, the boundary orientations do not match which contradicts Lemma 2.6. If the right 4-tile is a D-tile, we are in Case 4 and obtain a contradiction as explained before.

Therefore, both 4-tiles have to be I-tiles, i.e., we have3.8As in Case 4, we consider two 4-tiles attached on the top. By using arguments similar to the ones above, we will show that the only possible choice how to assemble the four 4-tiles would be given by3.9This, however, is excluded by Lemma 3.6(iv). To see (3.9), in view of the fact that we have already ruled out Cases 1–5 and the boundary orientations need to match by Lemma 2.6, only the 4-tiles3.10can be attached on top of the left I-tile in (3.8). Analogously, on top of the right I-tile in (3.8) we can only attach the 4-tiles3.11see Table 2. As in Case 4 we consider the middle 4-tile between the left I-tile in (3.8) and the 4-tile on top of it (left middle 4-tile) and the middle 4-tile between the right I-tile in (3.8) and the 4-tile on top of it (right middle 4-tile). In view of (3.10)–(3.11), there are only the cases indicated in Table 5. From Table 5 we see that the boundary orientations of the shared boundary of the two middle 4-tiles can only match if the right middle 4-tile is of type . By (3.11) this shows that only  can be attached on top of the right I-tile . Then, in view of (3.10), only  can be attached on top of the left I-tile  as the other four 4-tiles in (3.10) do not math the boundary orientation of . This shows that (3.9) holds, and concludes the proof of Case 6.

**Table 5:** The different possible middle 4-tiles in Case 6

Middle 4-tile left	Middle 4-tile right
	
	
	

*Step 2:* 4*-tiles on the diagonal.* We now show that in four adjacent 4-tiles arranged in a square, the two pairs of diagonal 4-tiles cannot roll-up/down along different diagonals. Up to interchanging the roles of $\bullet$ and $\circ$, and up to reflection along the $e_1$- or the $e_2$-axis, there are two cases to consider, where Case 1 represents one of the eight situationsand Case 2 represents one of the eight situationsHere, as in Step 1, the symbols $\bullet$ and $\circ$ indicate both the corresponding I-tile and D-tile. Without restriction we address only the first configuration in both cases as all other situations can be treated along similar lines.

*Case 1.* We start by introducing the labelingWe preliminarily note that, in view of Step 1, for ${\mathfrak {B}}$ and ${\mathfrak {D}}$ only 4-tiles in ${\mathcal {A}} \cap {\mathcal {B}}$ are admissible, see (3.2)–(3.3), i.e., the two Z-tiles  and . We distinguish three different subcases:

*Case 1.1.* If ${\mathfrak {A}}$ is the unique I-tile, then Lemma 2.6 for the boundary between ${\mathfrak {A}}$ and ${\mathfrak {D}}$ as well as the boundary between ${\mathfrak {D}}$ and ${\mathfrak {C}}$ implies that the 4-tile ${\mathfrak {D}}$ cannot be a Z-tile. In fact, the boundary orientation of ${\mathfrak {A}}$ on the right is $\wedge$ (indicated by < in the notation) and the boundary orientation of ${\mathfrak {C}}$ on top is $\vee$.

*Case 1.2.* By a similar reasoning, if ${\mathfrak {A}}$ is the unique D-tile and ${\mathfrak {C}}$ is the unique I-tile, Lemma 2.6 implies that the 4-tile ${\mathfrak {B}}$ cannot be a Z-tile.

*Case 1.3.* If both ${\mathfrak {A}}$ and ${\mathfrak {C}}$ are D-tiles, we again use Lemma 2.6 and see that the 4-tiles ${\mathfrak {B}}$ and ${\mathfrak {D}}$ can only be of type . Therefore, we need to consider the configurationThe middle 4-tile between ${\mathfrak {A}}$ and ${\mathfrak {B}}$ is given by  and the middle 4-tile between ${\mathfrak {C}}$ and ${\mathfrak {D}}$ is given by . Their shared boundary have mismatching boundary orientations, contradicting Lemma 2.6.

*Case 2.* We start by introducing the labelingAs in Case 1, due to Step 1, for ${\mathfrak {B}}$ and ${\mathfrak {D}}$ only the two Z-tiles  and  are admissible. We distinguish four different subcases:

*Case 2.1.* If both ${\mathfrak {A}}$ and ${\mathfrak {C}}$ are I-tiles, Lemma 2.6 implies that the 4-tile ${\mathfrak {B}}$ cannot be a Z-tile.

*Case 2.2.* If both ${\mathfrak {A}}$ and ${\mathfrak {C}}$ are D-tiles, then Lemma 2.6 implies that the 4-tile ${\mathfrak {B}}$ cannot be a Z-tile.

*Case 2.3.* If ${\mathfrak {A}}$ is the unique D-tile and ${\mathfrak {C}}$ is the unique I-tile, then Lemma 2.6 implies that the 4-tile ${\mathfrak {D}}$ cannot be a Z-tile.

*Case 2.4.* Now suppose that ${\mathfrak {A}}$ is the unique I-tile and ${\mathfrak {C}}$ is the unique D-tile. Then ${\mathfrak {B}}$ and ${\mathfrak {D}}$ need to be of type . Therefore, we need to consider the configuration3.12and show that it is also not admissible. The I-tile rolls up in direction top left, which has no influence in this (sub-)configuration. In other words, by replacing in (3.12) the tile ${\mathfrak {A}}$ with the Z-tile  and showing that this modified configuration is not admissible, we also find that (3.12) is not admissible. In fact, in view of Lemma 3.6(i) and the fact that the D-tile  lies in ${\mathcal {B}}$ (see (3.3)), we see that the modified version of (3.12) is not admissible. This concludes this step of the proof. $\square$

### Constant Type Along the Diagonal

This subsection is devoted to the proof of Proposition 3.4.

#### Proof of Proposition 3.4

We assume without restriction that all four 4-tiles lie in ${\mathcal {A}}$, see (3.2), as the other case is completely analogous. We considerand note that we need to show that ${\mathfrak {B}}$ and ${\mathfrak {D}}$ are of the same type. We proceed in two steps: first, we show that ${\mathfrak {B}}$ and ${\mathfrak {D}}$ are of the same *class,* i.e., both have to be either Z-, I-, or D-tiles. In the second step, we then conclude that they even have to be of the same *type*. In the proof, we will use the following observation which directly follows from the definition of ${\mathcal {A}}$:

3.13 $$\begin{aligned} \begin{aligned}&\bullet \,\text {Z- and D-tiles:}&\text {All four boundary orientations are identical}, \\&\bullet \,\text {I-tiles:}&\text {Left and upper boundary orientations are identical,} \\&\text {right and lower boundary orientations are identical}. \end{aligned} \end{aligned}$$

*Step 1.* In this step, we show that ${\mathfrak {B}}$ and ${\mathfrak {D}}$ are necessarily of the same class.

*Case 1.1*. If exactly one of the two tiles ${\mathfrak {B}}$ and ${\mathfrak {D}}$ is an I-tile, in view of (3.13), we obtain a contradiction to Lemma 2.6 as not all boundary orientations of the four shared boundaries can match.

Thus, we can now assume that none of the tiles ${\mathfrak {B}}, {\mathfrak {D}}$ is an I-tile. Actually, it is also not restrictive to assume that the tiles ${\mathfrak {A}}$ and ${\mathfrak {C}}$ are not of class I. Indeed, the upper left optimal cell of ${\mathfrak {A}}$ and the lower right optimal ${\mathfrak {C}}$ have no influence on the subsequent arguments in Cases 1.2–1.4 and can readily be replaced by the other type. This allows to replace tiles of class I by types of class Z or D in ${\mathcal {A}}$, without affecting the following arguments. Summarizing, it suffices to consider the case that all four 4-tiles are Z- or D-tiles.

*Case 1.2*. If three 4-tiles are D-tiles and one tile is a Z-tile, we only have that ${\mathfrak {B}}$ and ${\mathfrak {D}}$ are not of the same class if the Z-tile lies in $\lbrace {\mathfrak {B}}, {\mathfrak {D}} \rbrace$. This contradicts Lemma 3.6(iii).

*Case 1.3*. If three 4-tiles are Z-tiles and one tile is a D-tile, we only have that ${\mathfrak {B}}$ and ${\mathfrak {D}}$ are not of the same class if the D-tile lies in $\lbrace {\mathfrak {B}}, {\mathfrak {D}} \rbrace$. This contradicts Lemma 3.6(i).

*Case 1.4*. If two 4-tiles are of class Z and two of class D, the claim follows directly from Lemma 3.6(ii).

*Step 2.* In this second step we show that not only the class but also the type has to be constant along the diagonal. First, if we had different Z-tiles or D-tiles along the diagonal, in view of (3.2), these two 4-tiles would have different boundary orientations. Again by using (3.13), we obtain a contradiction to Lemma 2.6 as not all boundary orientations of the four shared boundaries can match.

We now address the case that ${\mathfrak {B}}$ and ${\mathfrak {D}}$ are I-tiles. Again in view of (3.13) and the definition of ${\mathcal {A}}$, we findsince otherwise the boundary orientations do not match, contradicting Lemma 2.6. Whenever the type is not constant along the diagonal, the 4-tile in the middle of the four 4-tiles is an A-tile which contradicts Proposition 3.2. For simplicity, we show this only in case a) as case b) follows along similar lines. In fact, by Lemma 2.6 we find that ${\mathfrak {A}}$ can only be of type , , , or , and ${\mathfrak {C}}$ can only be of type , , , or . Consequently, if ${\mathfrak {B}}$ is of type , in the middle we find the A-tile  or , and if ${\mathfrak {B}}$ is of type , we find the A-tile  or , see Table 1. $\square$

### Admissible Arrangement of 4-Tiles

This subsection is devoted to the proof of Proposition 3.5.

#### Proof of Proposition 3.5

Without restriction we perform the proof only for the types ${\mathcal {A}}$ defined in (3.2).

(i) We start by observing that each pair of 4-tiles in ${\mathcal {A}}$ with matching boundary orientations can be attached since all boundary angles are either Z- or D-boundary angles, see Table 3 and Table 1, and both angles coincide with $\delta _\theta$, see Lemma 3.1. We first show that the 4-tile in the middle is again in ${\mathcal {A}}$. In a second step, we check that the middle 4-tile is also coplanar.

We recall that the type of the middle 4-tile can by determined by considering the matrix notation, as exemplified in (2.22). In view of (3.2), we obtain the following six cases:

*Case 1.* Attaching two Z-tiles, we find that the two tiles are of same type and the middle tile is the Z-tile of the other type.

*Case 2.* Attaching two D-tiles, we find that the two tiles are of same type and the middle tile is again of this type.

*Case 3.* Attaching two I-tiles, we can obtain all possible 4-tiles in ${\mathcal {A}}$.

*Case 4.* Attaching a Z- and a D-tile, we obtain an I-tile in ${\mathcal {A}}$.

*Case 5.* Attaching a Z- and an I-tile, we obtain any Z- and I-tile in ${\mathcal {A}}$.

*Case 6.* Attaching a D- and an I-tile, we obtain any D- and I-tile in ${\mathcal {A}}$.

Note that in all cases above exactly 4-tiles from ${\mathcal {A}}$ can occur, and no more than those.

It remains to show that the resulting middle 4-tile is also coplanar. As attaching two 4-tiles does not change the optimal angle $\theta$, also the middle 4-tile consists of four optimal cells with angle $\theta$. Therefore, relation (2.19) holds for the middle 4-tile as well. To conclude the proof, it suffices to show that one of the nonplanarity angles $\delta _{13}$ and $\delta _{24}$ of the middle 4-tile is equal to $\delta _\theta$. To this end, note that one of these angles coincides with the boundary angle of the shared boundary of the two 4-tiles. By Lemma 3.1 this angle is equal to $\delta _\theta$.

**Fig. 7:**
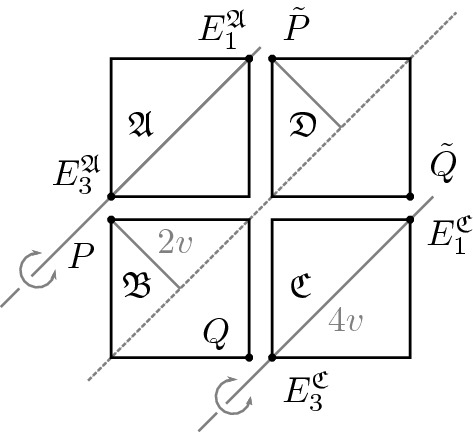
The points and rotations indicated in the proof of Proposition 3.5

(ii) We proceed constructively to show that every configuration consisting of four 4-tiles from ${\mathcal {A}}$ arranged in a square satisfying the matching conditions (M1)–(M2) is admissible, i.e., can be realized by an admissible deformation *y*. By assumption, ${\mathfrak {B}}$ and ${\mathfrak {D}}$ are of the same type. Then, one can check that, for any choice of ${\mathfrak {A}}, {\mathfrak {C}} \in {\mathcal {A}}$ satisfying the matching conditions (M1)–(M2), the boundary orientations of ${\mathfrak {A}}, {\mathfrak {C}}$ match with those of ${\mathfrak {B}}$ and ${\mathfrak {D}}$. In view of Lemma A.1(i), fixing ${\mathfrak {B}}$ in reference configuration and translating ${\mathfrak {D}}$ from its reference position by the vector (2*s*, 2*s*, 0), we see that these two 4-tiles share exactly one corner point, and we have $\vert P - {{\tilde{P}}} \vert = \vert Q - {{\tilde{Q}}} \vert = \sqrt{(2s)^2 + (2s)^2} = 4v$, where $P, Q \in {\mathfrak {B}}$ and ${{\tilde{P}}}, {{\tilde{Q}}} \in {\mathfrak {D}}$ are the corner vertices indicated in Fig. 7. By Lemma A.1(i) the opposite corner points along the diagonal $d_1$ have distance 4*v*, i.e., $\vert E_1^{\mathfrak {A}} - E_3^{\mathfrak {A}} \vert = \vert E_1^{\mathfrak {C}} - E_3^{\mathfrak {C}} \vert = 4v$. Therefore, we can translate ${\mathfrak {A}}$ and ${\mathfrak {C}}$ from their reference positions such that their opposite corner points coincide with *P* and ${{\tilde{P}}}$ and *Q* and ${{\tilde{Q}}}$, respectively. Since, for every 4-tile the distance between its center and a corner point equals $\sqrt{s^2+s^2} = 2v$, see (2.16) and Lemma 2.5(i), after rotating ${\mathfrak {A}}$ and ${\mathfrak {C}}$ about $(0,2s,0) + {\mathbb {R}}(1,1,0)$ and $(2s,0,0) + {\mathbb {R}}(1,1,0)$, respectively, as indicated in Fig. 7, the corner points of ${\mathfrak {A}}$, ${\mathfrak {B}}$, ${\mathfrak {C}}$, and ${\mathfrak {D}}$ in the interior of the configuration coincide. As the boundary orientations match by (M1)–(M2) and the boundary angles coincide by Lemma 3.1, also the respective middle points coincide after rotation of ${\mathfrak {A}}$ and ${\mathfrak {C}}$. This along with part (i) of the statement shows that the configuration is indeed realizable by an admissible configuration $y:\lbrace 0, 1,2,3,4\rbrace ^2 \rightarrow {\mathbb {R}}^3$. This concludes the proof. $\square$

## Figures and Tables

**Fig. 8:**
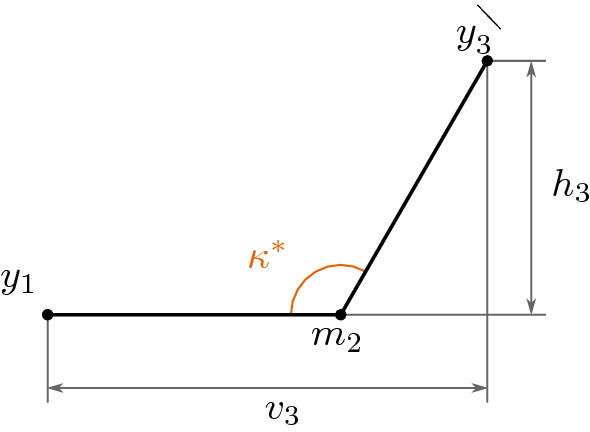
Cross section along the plane spanned by *a* and *n*, as defined in the proof of Lemma 2.3. From this perspective the points $y_2$, $m_2$, and $y_4$ coincide

**Fig. 9:**
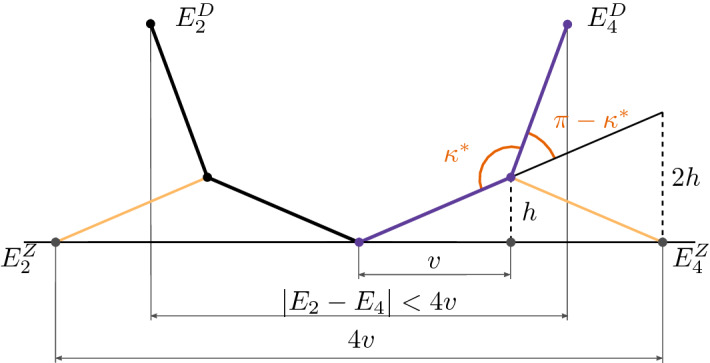
Cross section of a D-tile (bold, black and purple) and of a Z-tile (thin lines in light orange), positioned as in Lemma A.1. The distance between the diagonals is 4*v* for the Z-tile and smaller for the D-tile

**Fig. 10:**
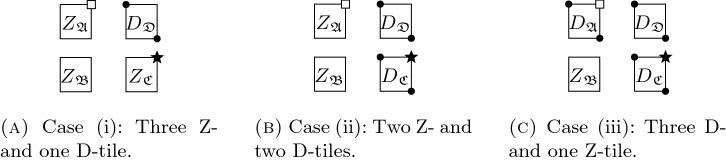
Different cases in the proof of Lemma 3.6

**Fig. 11:**
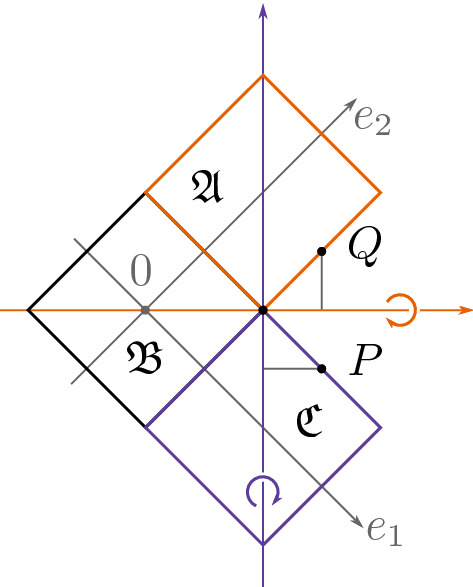
The points *P* and *Q* are rotated by $2\kappa$ around the axis in the respective directions. Note that *P* rotates clockwise

**Fig. 12:**
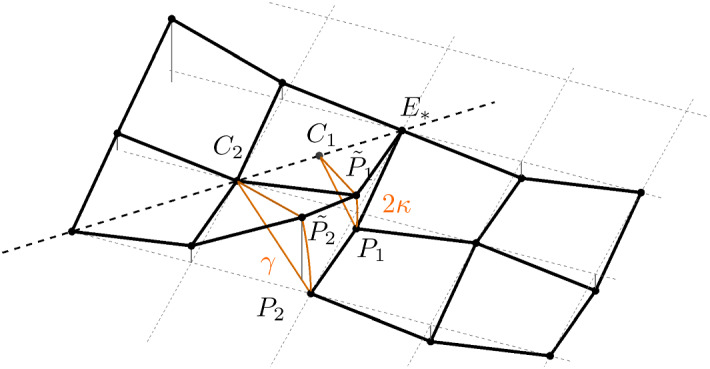
Rotation of a Z-tile around the diagonal of a D-tile in order to match the boundaries

**Fig. 13:**
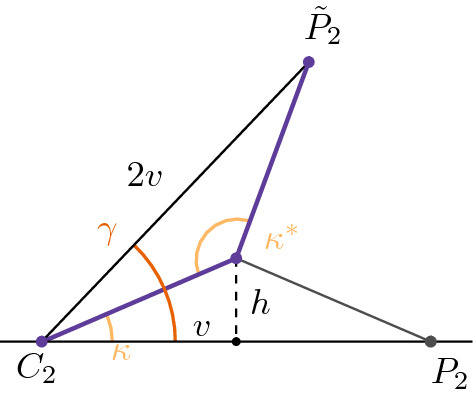
Cross section along the plane with normal (1, 1, 0)

**Fig. 14:**
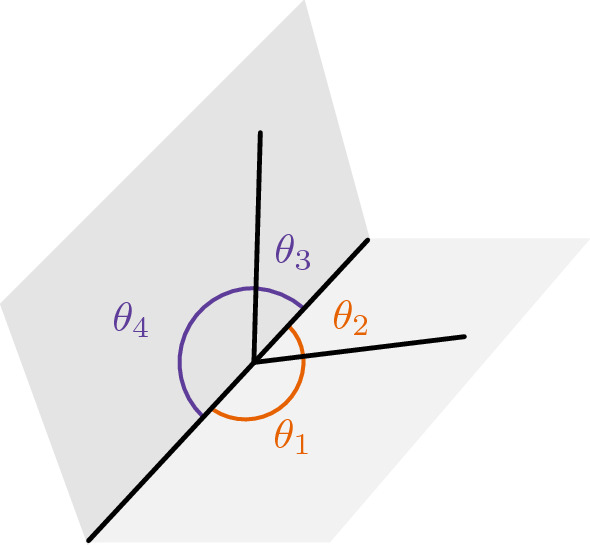
If $\delta _{13} = \pi$, then $\theta _1 + \theta _2 = \theta _3 + \theta _4 = \delta _{13} = \pi$ and thus $\theta _1 + \cdots + \theta _4 = 2\pi$. Furthermore, the angle $\delta _{24}$ can be chosen arbitrarily

## Appendix A: Remaining Proofs

### Geometry of Optimal Cells and 4-Tiles

This subsection is devoted to the proofs of the lemmas stated in Sect. 2.3.

#### Proof of Lemma 2.3

Recall that $m_2 :=(y_2 + y_4)/2$ is the middle point between $y_2$ and $y_4$, cf. Fig. 2. We define $a = m_2 - y_1$, with $\vert a \vert = d$. Let *n* be a normal vector to the plane spanned by $y_1$, $y_2$, and $y_4$, in direction $(y_2 - y_1) \times (y_4 - y_1)$.

Observe that by assumption the fourth point $y_3$ has to satisfy $\vert y_2 - y_3 \vert = \vert y_4 - y_3 \vert = 1$ and thus has to lie on the plane spanned by *a* and *n*. Therefore, we can make the ansatz

$$\begin{aligned} y_3 = y_1 + v_3 \, a \pm h_3\, n, \end{aligned}$$

where $$v_3$$ and $$h_3$$ are to be determined, see Fig. 8. Note that in ± we choose $$+$$ for form $$\diagdown $$ and − for form $$\diagup $$ . To conclude, we are left to prove that $$v_3$$ and $$h_3$$ can be determined uniquely. Since the cell is optimal, we have $$\measuredangle y_3\, m_2\, y_1 = \kappa ^*$$ (see (2.17)) as well as $$\vert a \vert = \vert m_2 - y_1 \vert = |m_2 - y_3 | = d$$. Consequently, the triangle with vertices $$ y_1$$, $$m_2$$, and $$y_3$$ and thus also the values of $$v_3$$ and $$h_3$$ are uniquely determined. $$\square $$

For convenience, we proceed with the proof of Lemma 2.5 and show Lemma 2.4 afterwards.

#### Proof of Lemma 2.5

In the proof, we again use the notation indicated in Fig. 5. We recall the definition in (2.18) and drop for the moment the condition $\delta _{13} = \delta _{24}$ induced by (2.14). To verify that every 4-tile can be placed in reference position, we first rotate and translate the 4-tile such that $C=0$ and $M_1 = (s_1,0,h_1)$, and $M_3 = (-s_1,0,h_1)$, where a simple trigonometric relation yields

A.1 $$\begin{aligned} s_1 = \cos \left( \frac{\pi - \delta _{13}}{2} \right) = \sin (\delta _{13}/2), \quad h_1 = \sin \left( \frac{\pi -\delta _{13}}{2} \right) = \cos (\delta _{13}/2). \end{aligned}$$

Here, we note that $s_1>0$, while $h_1$ is negative whenever $\delta _{13} > \pi$. We now show that the coordinates of $M_2$ and $M_4$ are given by

A.2 $$\begin{aligned} M_2 = (0,s_2, h_2), \quad \quad \quad M_4 = (0, -s_2, h_2), \end{aligned}$$

where $s_2= \sin (\delta _{24}/2)$ and $h_2 = \cos (\delta _{24}/2)$. We focus on $M_2$ since the argument for $M_4$ is analogous. For convenience, we write $M_2 = (p_1,p_2,p_3)$ and use the definition of optimal cells, i.e., $\measuredangle M_1 \, C \, M_2 = \theta = \measuredangle M_2 \, C \, M_3$ and $|M_1| = |M_2| = |M_3| = 1$, to find

$$\begin{aligned} \cos \theta = M_1 \cdot M_2 = p_1 s_1 + p_3 h_1, \quad \quad \cos \theta = M_3\cdot M_2 = - p_1 s_1 + p_3 h_1. \end{aligned}$$

By combining the two equalities we get $p_1 = 0$. In view of (A.1), $p_3$ is then given by

A.3 $$\begin{aligned} p_3 = \frac{\cos \theta }{h_1} = \frac{\cos \theta }{\cos (\delta _{13}/2)}, \end{aligned}$$

and, since $|M_2| = 1$, we find $p_2 = \sqrt{1-p_3^2}$. Thus, we have $M_2 =(0,p_2,p_3)$. By a similar argument we find $M_4 = (0,-p_2,p_3)$. To conclude for (A.2), we need to find the relation between $p_2$ and $p_3$. To this end, we use the fact that $\measuredangle M_2 \, C \, M_4 = \delta _{24}$ to calculate $\cos (\delta _{24}) = M_2 \cdot M_4 = p_3^2 - p_2^2$. This, together with $p_2^2+p_3^2 = 1$, verifies that $p_3 = \sqrt{( 1+\cos (\delta _{24}) )/2} = \cos (\delta _{24}/2)$ by using the double-angle formula. Correspondingly, we find $p_2 = \sin (\delta _{24}/2)$. This proves (A.2). Let us remark for later purposes that (A.3) implies

A.4 $$\begin{aligned} \cos (\delta _{13}/2) \cos ( \delta _{24}/2) = \cos \theta . \end{aligned}$$

From the condition $\delta _{13}=\delta _{24}$ we get that $s=s_1=s_2$ and $h=|h_1|=|h_2| = \sqrt{1-s^2}$. We also let $\varsigma = \mathrm{sgn}(h_1) = \mathrm{sgn}(h_2)$. To conclude the proof of (i), it remains to check that $s = \sqrt{2}v$, where *v* is defined in (2.16), i.e., is chosen in such a way that 2*v* indicates the length of a diagonal in an optimal cell. This length can indeed be expressed as $|M_i-M_{i+1}| = \sqrt{2}s$ for $i=1,\ldots ,4$, which yields the desired relation.

We proceed with the proof of (ii). By fixing $\theta$, the angle $\delta _\theta$ is also determined and, by (i), also fixing $\mathrm{sgn}(h_1)$ determines completely the positions of the points $(M_i)_{i=1}^4$. In view of Lemma 2.3, the positions of $(E_i)_{i=1}^4$ are determined as well, as soon as the forms of the four optimal cells are given. $\square$

#### Proof of Lemma 2.4

In the proof of Lemma 2.5 we have already verified (2.19), see (A.4). Consider $f_\theta :[0,\pi ]\rightarrow {\mathbb {R}}$ defined by

$$\begin{aligned} f_\theta (\delta ) = 2\arccos (\cos \theta /\cos (\delta /2)). \end{aligned}$$

As $\cos \theta >0$, $f_\theta$ is decreasing and thus has at most one fixed point. Hence, $f_\theta$ has exactly one fixed point given by $\delta _\theta = 2 \arccos (\sqrt{\cos \theta })$. This eventually shows that $\delta _{13}$ and $\delta _{24}$ coincide if and only if $\delta _{13} = \delta _{24} = \delta _\theta$. $\square$

We close this subsection with an elementary observation. We again refer to the notation in Fig. 5.

#### Lemma A.1

(i) For any coplanar 4-tile in ${\mathcal {A}}$ (cf. (3.2)) in reference position, see Lemma 2.5(i), we have $E_1 = (s,s,0)$ and $E_3= (-s,-s,0)$.

(ii) For any Z-tile in ${\mathcal {A}}$ in reference position, we have $E_2 = (-s,s,0)$ and $E_4= (s,-s,0)$.

(iii) For any D-tile in ${\mathcal {A}}$ in reference position, we have $|E_2-E_4| < 4v$, where *v* is given in (2.16).

(iv) Assume that an optimal cell $\{y_1,\dots , y_4\}$ is positioned in such a way that $e_3 \cdot y_1 = 0$ and $e_3 \cdot y_2 = e_3 \cdot y_4 = h$. Then, depending on its form, we have $e_3 \cdot y_3 = 0$ or $e_3 \cdot y_3 > 2h$.

Similar statements as (ii)–(iv) hold for ${\mathcal {B}}$ in place of ${\mathcal {A}}$ by changing the roles of the diagonals.

#### Proof

Without restriction, we consider a 4-tile in ${\mathcal {A}}$ in reference position such that $\varsigma = 1$, cf. Lemma 2.5, as the other case only amounts to reflection along the $e_1$-$e_2$-plane. By Lemma 2.5(i) we have that $M_1 = (s,0,h)$, $M_2 = (0,s,h)$, $M_3 = (-s,0,h)$, and $M_4 = (0,-s,h)$. The optimal cells $\{C, M_1, E_1, M_2 \}$ and $\{C, M_3, E_3, M_4\}$ are of form $\diagup$, see Fig. 2 and (3.2). Thus, by Lemma 2.3 we get $E_1 = (s,s,0)$ and $E_3 = (-s,-s,0)$. This shows (i). We now suppose that the 4-tile is either of class Z or of class D, i.e., is of type  or . Therefore, the two optimal cells $\{C, M_2, E_2, M_3 \}$ and $\{C, M_4, E_4, M_1\}$ are of form $\diagup$ (D-tile) and of form $\diagdown$ (Z-tile), which yields to a cross section along the direction $(-1,1)$ as indicated in Fig. 9. We now obtain

A.5 $$\begin{aligned}&E_2^D \cdot e_3 = E_4^D \cdot e_3 > 2h \qquad \text {for the D-tile and}\nonumber \\&E_2^Z \cdot e_3 = E_4^Z \cdot e_3 =0 \qquad \quad \text {for the Z-tile}. \end{aligned}$$

Indeed, for the Z-tile this follows from Lemma 2.3. For the D-tile we use Thales’ intercept theorem instead, with reference to Fig. 9. In particular, this implies (ii). Then, as in the Z-cell the distance of the diagonals is $4v = 2\sqrt{2}s$, (A.5) and Fig. 9 show that in the D-cell we have $\vert E_2^D - E_4^D \vert < 4v$. This implies (iii). Eventually, property (iv) follows from (A.5). $\square$

### Boundary Orientations and Attachment of Two 4-Tiles

This subsection is devoted to the proofs of Lemma 2.6 and Lemma 2.7.

#### Proof of Lemma 2.6

The statement for the boundary orientation and the boundary angle, defined in (2.20)–(2.21), respectively, follows from the fact that the notions are determined uniquely by the three points which are shared by the two 4-tiles. More precisely, given any 4-tile in reference position, by applying a rotation about the $e_3$ axis composed with a further small rotation (depending on $\theta$), and a translation one can ensure that a boundary of the 4-tile is contained in the $e_2$-$e_3$-plane and is symmetric with respect to the $e_1$-$e_3$-plane. Provided that $\theta$ is small, one can check that this transformation does not change the inequality in (2.20). Clearly, each two 4-tiles with the same boundary angles can be transformed in this fashion in order to be matched along the shared boundary.

Consider now two attached 4-tiles positioned such that the middle 4-tile is in reference position, in particular, the shared middle point of the boundary is the origin. If the boundary orientation of the shared boundary is $\wedge$, then both shared corner vertices satisfy $E_{i-1} \cdot e_3, \, E_i \cdot e_3 < 0$, see (2.20), and thus for the middle 4-tile we have $\varsigma = -1$. An analogous argument applies if the boundary is $\vee$. $\square$

#### Proof of Lemma 2.7

First, we note that, for any 4-tile in reference position, reflection about the $e_1$-$e_2$-plane interchanges all boundary orientations since the reflection changes the sign of any $e_3$-component. Moreover, rotation around $e_3$ by $\pi /2$ leaves the boundary orientation invariant. A rotation in the matrix notation therefore simply rotates the corresponding sides and interchanges $\vee$ with > and $\wedge$ with <. For example, rotating  by $\pi /2$ counterclockwise, yields . This entails that it is enough to check the boundary orientations for one representative of any class in Table 2.

First, by Lemma 2.5 and Lemma A.1 we get that the orientation of all boundaries of the coplanar D-tile  is $\vee$. Indeed, assume that the 4-tile is in reference position and use the notation of Fig. 5. Then the corner vertices $E_1 \cdot e_3 = E_3\cdot e_3 = 0$ and $M_i \cdot e_3 = h$ for $i=1,\ldots ,4$. Moreover, the optimal cells $\{C, M_2, E_2, M_3\}$ and $\{C, M_4, E_4, M_1\}$ are positioned as in Lemma A.1(iv). Thus, we can conclude that the corner vertices $E_2$ and $E_4$ have $e_3$-coordinate strictly larger than 2*h* and hence, in view of (2.20), we find that the boundary orientation is $\vee$.

Consider the Z-tile  in reference position. In this case, the middle points satisfy $M_i \cdot e_3 = h$, $i = 1, \dots , 4$ and, in view of the forms of the four optimal cells, the corner points satisfy $E_i \cdot e_3 = 0$, $i = 1,\dots , 4$. Thus, all four boundaries have orientation $\wedge$.

We observe that the above arguments actually only take into account the relative position of the two optimal cells adjacent to a boundary. Thus, one can repeat the arguments above for the I-tiles. For instance,  has two $\vee$ boundaries top and left, i.e., adjacent to $\bullet$ as in a D-tile, and two $\wedge$ boundaries right and bottom, as in a Z-tile. $\square$

### Arrangements of Four 4-Tiles

In this subsection we prove Lemma 3.6. We start by a result about the mutual position of two attached 4-tiles. To this end, recall the types of 4-tiles ${\mathcal {A}}$ and ${\mathcal {B}}$ introduced in (3.2)–(3.3), as well as the definition of *s* and *h* in Lemma 2.5(i).

#### Lemma A.2

Let *T* and ${{\tilde{T}}}$ be two attached Z-, I-, or D-tiles of an admissible configuration. Without restriction, up to applying an isometry, suppose that *T* is in reference position, and that the shared boundary consists of the three points $E_1$, $M_1$, $E_{4}$ and ${{\tilde{E}}}_2$, ${{\tilde{M}}}_2$, ${{\tilde{E}}}_{3}$, respectively, referring to the notation in Fig. 5. We denote the reference position corresponding to ${{\tilde{T}}}$ by ${{\tilde{T}}}'$. We denote by $R^{{\mathcal {A}}}_{\alpha }$ the counterclockwise rotation around the axis (1, 1, 0) by the angle $\alpha$, and $R^{{\mathcal {B}}}_{\alpha }$ denotes the counterclockwise rotation around the axis $(-1,1,0)$ by the angle $\alpha$.

(1) If the shared boundary is a Z-boundary of *T*, and a Z-boundary of ${{\tilde{T}}}$, then we have ${{\tilde{T}}} = (2s,0,0)+ {{\tilde{T}}}'$.

(2) If the shared boundary is a D-boundary of *T* and a Z-boundary of ${{\tilde{T}}}$, we have

$$\begin{aligned} {{\tilde{T}}} = {\left\{ \begin{array}{ll} R^{{\mathcal {A}}}_{2\varsigma _T\kappa } ( (2s,0,0)+ {{\tilde{T}}}') &{} \quad \text { if }\, T \in {\mathcal {A}}, \\ R^{{\mathcal {B}}}_{2\varsigma _T\kappa }( (2s,0,0) + {{\tilde{T}}}') &{} \quad \text { if}\, T \in {\mathcal {B}}, \end{array}\right. } \end{aligned}$$

where $\kappa$ is defined in (2.17), and $\varsigma _T$ corresponding to *T* is given in Lemma 2.5.

(3) If the shared boundary is a Z-boundary of *T* and a D-boundary of ${{\tilde{T}}}$, we have

$$\begin{aligned} {{\tilde{T}}} = {\left\{ \begin{array}{ll} (2s,0,0)+ R^{{\mathcal {A}}}_{2\varsigma _{{{\tilde{T}}}'}\kappa } {{\tilde{T}}}' &{}\quad \text { if}\, {{\tilde{T}}} \in {\mathcal {A}}, \\ (2s,0,0)+ R^{{\mathcal {B}}}_{2\varsigma _{{{\tilde{T}}}'}\kappa } {{\tilde{T}}}' &{}\quad \text { if }\, {{\tilde{T}}} \in {\mathcal {B}}, \end{array}\right. } \end{aligned}$$

where $\varsigma _{{{\tilde{T}}}'}$ corresponds to ${{\tilde{T}}}'$.

The case of two shared D-boundaries is not addressed here as we will not need it in the sequel. We warn the reader that, in the applications below without further mentioning, we will apply isometries to the tiles in order to reduce the positions to the ones indicated in the lemma. We postpone the proof of Lemma A.2 to the end of this subsection, and proceed with the proof of Lemma 3.6.

#### Proof of Lemma 3.6

(i) Without restriction we suppose that the tiles lie in ${\mathcal {A}}$ and we suppose by contradiction that the D-tile is given by ${\mathfrak {D}}$. We assume that ${\mathfrak {B}}$ is given in reference position. Then, by Lemma A.2(1) we see that ${{\mathfrak {C}}}$ is in reference position shifted by (2*s*, 0, 0), and ${{\mathfrak {A}}}$ is in reference position shifted by (0, 2*s*, 0). By Lemma 2.5 this implies that the coordinates of the points *Q* and *P*, indicated with $\square$ and respectively $\star$ in Fig. 10A, are given by $Q = (s,3s,0)$ and $P = (3s,s,0)$, respectively. In particular, we have that $|P-Q| = 2\sqrt{2}s = 4v$, cf. (2.16) and Lemma 2.5(i), which corresponds to the length of the diagonal in ${\mathfrak {D}}$. For the D-tile ${\mathfrak {D}}$ in ${\mathcal {A}}$, however, having the rolling direction as given in Fig. 10A, cf. (3.2), the corresponding diagonal has length smaller than 4*v* by Lemma A.1(iii), a contradiction.

(ii) Without restriction we suppose that the tiles belong to ${\mathcal {A}}$ and we suppose by contradiction that the Z-tiles are in ${\mathfrak {A}}$, ${\mathfrak {B}}$, and that the D-tiles are in ${\mathfrak {C}}$, ${\mathfrak {D}}$, as in Fig. 10B. We also assume that ${\mathfrak {B}}$ is given in reference position. By Lemma A.2(1) we see that ${\mathfrak {A}}$ is in reference position shifted by (0, 2*s*, 0), and thus the point *Q*, indicated by $\square$, has coordinates $Q = (s,3s,0)$. By Lemma A.2(3) the position of the tile ${\mathfrak {C}}$ is obtained by taking the tile in reference configuration, rotating around the axis (1, 1, 0) by the angle $\pm 2\kappa$, and then by a shifting by (2*s*, 0, 0). As the corners where no roll-up occurs are left invariant under the rotation, we find by Lemma A.1(i) that the point *P*, denoted by a $\star$ in Fig. 10B, has coordinates (3*s*, *s*, 0). This implies $|P-Q| = 2\sqrt{2}s = 4v$. As in (i), this contradicts Lemma A.1(iii) since the length of the diagonal in the D-tile ${\mathfrak {D}}$ where the tile rolls-up is less than 4*v*.

(iii) Again without restriction we assume that the tiles belong to ${\mathcal {A}}$ and we suppose by contradiction that the Z-tile is in ${\mathfrak {B}}$, as in Fig. 10C. We assume that ${\mathfrak {B}}$ is given in reference position. By Lemma A.2(3) the position of ${\mathfrak {C}}$ is obtained by taking the tile in reference configuration, rotation around the axis (1, 1, 0) by the angle $\pm 2\kappa$, and then by a shifting by (2*s*, 0, 0) (exactly in this order). As the corners where no roll-up occurs are left invariant under the rotation, we find by Lemma A.1(i) that the point *P*, marked with $\star$ in Fig. 10C, has coordinates $P = (3s,s,0)$. In a similar fashion, the position of ${\mathfrak {A}}$ is obtained by taking the tile in reference configuration, rotating around the axis (1, 1, 0) by the angle $\pm 2\kappa$, and then by a shifting by (0, 2*s*, 0). Lemma A.1(i) yields that the point *Q*, indicated with $\square$ in Fig. 10C, has coordinates $Q= (s,3s,0)$. This implies $|P-Q| = 2\sqrt{2}s = 4v$, which as in (i) and (ii) contradicts Lemma A.1(iii) since the length of the diagonal in the D-tile ${\mathfrak {D}}$ where the tile roll-up is less than 4*v*.

(iv) We finally show that (3.6) is not admissible. As before, we denote the 4-tiles by ${\mathfrak {A}}, \ldots , {\mathfrak {D}}$, as indicated in (3.5). Our strategy hinges on (i)–(iii): we denote by ${{\tilde{Q}}}$ the right middle point of ${\mathfrak {A}}$ and by ${{\tilde{P}}}$ the upper middle point of ${\mathfrak {C}}$. In view of Lemma 2.5(i) applied on ${\mathfrak {D}}$, their distance necessarily needs to be $\sqrt{2}s = 2v$. We will show, however, that this is impossible.

In order to do so, we first assume that ${\mathfrak {B}}$ is in reference position. In view of Lemma A.2(3), the position of the tile ${\mathfrak {C}}$ is obtained by taking the tile in reference configuration, rotating it around the axis $(-1,1,0)$ by the angle $-2\kappa$, and then by a shifting by (2*s*, 0, 0) (exactly in this order). In a similar fashion, by Lemma A.2(2) the position of the tile ${\mathfrak {A}}$ is obtained by taking the tile in reference configuration, followed by a translation by (0, 2*s*, 0), and then by rotation around the axis (1, 1, 0) by the angle $-2\kappa$ (exactly in this order).

We will now change the coordinate system to simplify the notational realization of the procedure: we suppose that the common vertex of all three 4-tiles lies in the origin and we reorient the coordinate system such that the rotation axis (1, 1, 0) coincides with $e_1$ and the rotation axis $(-1,1,0)$ with $e_2$, see Fig. 11. Then, the points ${{\tilde{Q}}}$ and ${{\tilde{P}}}$ are given by ${{\tilde{Q}}} = {\mathcal {R}}^{e_1}_{2\kappa } Q$ and ${{\tilde{P}}} = {\mathcal {R}}^{-e_2}_{2\kappa } P = {\mathcal {R}}^{e_2}_{-2\kappa } P$, where $Q = (v,v,-h)$ and $P = (v,-v,-h)$ are calculated by using Lemma 2.5, and the rotations are given by

$$\begin{aligned} {\mathcal {R}}^{e_1}_{2\kappa } = \left( \begin{array}{ccc} 1 &{}\quad 0 &{}\quad 0 \\ 0 &{}\quad \cos (2\kappa ) &{}\quad -\sin (2\kappa ) \\ 0 &{}\quad \sin (2\kappa ) &{}\quad \cos (2\kappa ) \end{array} \right) \! , \ \ {\mathcal {R}}^{e_2}_{-2\kappa } = \left( \begin{array}{ccc} \cos (2\kappa ) &{}\quad 0 &{}\quad \sin (2\kappa ) \\ 0 &{}\quad 1 &{}\quad 0 \\ -\sin (2\kappa ) &{}\quad 0 &{}\quad \cos (2\kappa ) \end{array} \right) . \end{aligned}$$

An elementary calculation yields

$$\begin{aligned} {{\tilde{Q}}} = \left( \begin{array}{c} v \\ v \\ h \end{array} \right) \! , \ \ {{\tilde{P}}} = \left( \begin{array}{c} 0 \\ -v \\ 0 \end{array} \right) + {\mathcal {R}}^{e_2}_{-2\kappa } \left( \begin{array}{c} v \\ 0 \\ -h \end{array} \right) = \left( \begin{array}{c} 0 \\ -v \\ 0 \end{array} \right) + {\mathcal {R}}^{e_2}_{-4\kappa } \left( \begin{array}{c} v \\ 0 \\ h \end{array} \right) \! , \end{aligned}$$

where we used the definition of $\kappa = \arctan (h/v)$, see (2.17), and the trigonometric identities $\cos (2 \arctan (x)) = (1-x^2)/(1+x^2)$ and $\sin (2 \arctan (x)) = 2x/(1+x^2)$, as well as $(v,0,-h) = {\mathcal {R}}^{e_2}_{-2\kappa }(v,0,h)$. Consequently, we obtain

$$\begin{aligned} \vert {{\tilde{Q}}} - {{\tilde{P}}} \vert ^2 = (2v)^2 + \left| \big ({\mathcal {R}}^{e_2}_{0} - {\mathcal {R}}^{e_2}_{-4\kappa } \big ) (v,0,h) \right| ^2 \end{aligned}$$

which is strictly larger than $(2v)^2$ since $\kappa \in (0,\pi /2)$, see (2.17). This establishes a contradiction since, as stated above, the distance should be 2*v*. $\square$

#### Proof of Lemma A.2

(1) Since the shared boundary is a Z-boundary of *T* and a Z-boundary of ${{\tilde{T}}}$, and the boundary orientations of *T* and ${{\tilde{T}}}$ match at the shared boundary (see Lemma 2.6), Tables 2 and 3 show that $\varsigma _T = \varsigma _{{{\tilde{T}}}}$ and that the middle 4-tile between *T* and ${{\tilde{T}}}$, denoted by $T_*$, is a Z-tile. By Lemma 2.6 we also find $\varsigma _{T_*} = - \varsigma _T$. Then by Lemma A.1(ii) it is elementary to check that $C_{{{\tilde{T}}}} - C_T = (2s,0,0)$, where $C_{{{\tilde{T}}}}$ and $C_T$ denote the centers of the 4-tiles, respectively.

(2) We prove the result only for the particular case of the two 4-tiles  and , as depicted in Fig. 12. In fact, the general case can be reduced to this situation by (a) replacing the optimal cells which are not adjacent to the shared boundary, as they do not affect the argument; and by (b) applying a suitable rotation or reflection.

Suppose that *T* is in reference position and denote the reference position of $${{\tilde{T}}}$$ by $${{\tilde{T}}}'$$. We define $${{\tilde{T}}}'':={{\tilde{T}}}' + (2s,0,0)$$. In view of Lemma A.1(i),(ii), we see that *T* and $${{\tilde{T}}}''$$ share exactly one corner point $$E_* = (s,s,0)$$ as depicted in Fig. 12. Clearly, the rotation around the axis (1, 1, 0) by $$2\kappa $$ leaves $$E_*$$ invariant. We need to show that under this rotation the points $$P_i$$, $$i=1,2$$, are mapped to $${{\tilde{P}}}_i$$, as depicted in Fig. 12. We denote by $$C_i$$, $$i=1,2$$, the two points on (1, 1, 0) which intersect the plane with normal vector (1, 1, 0) containing $$P_i$$ and $${{\tilde{P}}}_{i}$$. By Lemma 2.5 we find that $$C_1 = (s/2,s/2,0)$$ and $$C_2 = 0 $$. We need to check that

A.6 $$\begin{aligned} |C_i - P_i| = |C_i - {{\tilde{P}}}_i| \quad \text { and } \quad \measuredangle P_i \, C_i \, {{\tilde{P}}}_i = 2\kappa \quad \text { for i=1,2}. \end{aligned}$$

We first address $i=1$. By Lemma 2.5, we have $P_1 = (s,0,-h)$ and ${{\tilde{P}}}_1 = (s,0,h)$. We also note that $s = \sqrt{2}v$. This along with $C_1 = (s/2,s/2,0)$, $\kappa = \arctan (h/v)$ (see (2.17)), and the trigonometric identity $\cos (2 \arctan (x)) = (1-x^2)/(1+x^2)$ yields (A.6) by an elementary computation.

We now address $i=2$. As $C_2$ and ${{\tilde{P}}}_2$ form a diagonal of an optimal cell, see Fig. 12, by the definition before (2.16) we get $|C_2 - {{\tilde{P}}}_2| = 2v = \sqrt{2}s$. On the other hand, by Lemma 2.5, we find $P_2 = (s,-s,0)$ and therefore $|P_2 - C_2| = \sqrt{2}s$. This shows the first part of (A.6). To calculate the angle, we refer to the cross section in Fig. 13. Since this cross section is the one of an optimal cell in a rotated position, we can calculate the angle $\measuredangle P_2 \, C_2 \, {{\tilde{P}}}_2$ using the definition of $\kappa ^*$ in (2.17) and thus derive that $\gamma = \kappa + (\pi - \kappa ^*)/2 = \kappa + \pi /2 - (\pi - 2\kappa )/2 = 2 \kappa$. This concludes the proof.

The proof of (3) is similar to (2) by interchanging the roles of the 4-tiles. We omit the details. $\square$

We proceed with a simple consequence for boundary angles defined in (2.21).

#### Corollary A.3

The boundary angle of a D-boundary coincides the the boundary angle of a Z-boundary.

This immediately follows from Lemma A.2(ii). Indeed, if the statement was not true, one could not attach the two 4-tiles, as described in the previous proof.

### Incidence Angles in Coplanar 4-Tiles: Theorem 2.8 Implies Theorem 2.2

In this short subsection, we explain that Theorem 2.8 implies Theorem 2.2. In Sect. 2.5, we already addressed the type function. Therefore, it remains to consider the last two items in Theorem 2.2, i.e., the incidence angles defined in (2.15) between optimal cells.

*Z-tiles.* We start by showing that the incidence angles between optimal cells in a Z-tile along both diagonals are given by zero. Observe that reflection about the $e_1$-$e_2$-plane only interchanges the sign of the incidence angle. Thus, we assume without restriction that $\varsigma = 1$ (cf. Lemma 2.5). Due to symmetry, it suffices to consider one of the four bonds contained in the 4-tile, e.g., the bond connecting *C* and $M_1$, referring to the notation in Fig. 5. Without restriction we only calculate the incidence angle along $d_1$ as the other one can be calculated in a similar fashion, again exploiting symmetry. If the 4-tile is in reference position, Lemma 2.5(i) implies that $y_{\mathrm{top}}^1 = E_1 = (s,s,0)$, $y_{\mathrm{bot}}^1 = M_4 = (0,-s,h)$, $C=0$, and $M_1 = (s,0,h)$. Therefore, since $n^1_{\mathrm{top}}$ is in direction $M_1 \times E_1$ and $n^1_{\mathrm{bot}}$ is in direction $-M_1 \times (M_4-M_1)$, we find $n^1_{\mathrm{top}} = n^1_{\mathrm{bot}} = \frac{1}{\sqrt{s^2+2h^2}}(-h,h,s)$. Thus, in view of (2.15), we get that the incidence angle is $\arccos (1) = 0$.

*D-tiles.* We now address a D-tile in (2.23), given in reference configuration. Due to symmetry, it is again not restrictive to consider only the bond connecting *C* and $$M_1$$ and to suppose that $$\varsigma = 1$$. Note that, due to Lemma 2.5 and Lemma A.1(i), we have $$E_1 = (s,s,0)$$ and $$E_4$$ satisfies $$E_4 \cdot e_3 = q $$ and $$0< E_4 \cdot e_1 = - E_4 \cdot e_2 = p:= \sqrt{s^2-q^2/2}< s$$ for some $$q >0$$. Since $$E_1$$, $$M_1$$, and $$M_4$$ have the same position as in a Z-tile, repeating the above calculation we find that the incidence angle along $$d_1$$ is zero. We now consider the angle along $$d_2$$. To this end, we first find that $$ y_{\mathrm{top}}^2 = M_2 = (0,s,h) $$ and $$ y_{\mathrm{bot}}^2 = E_4 = (p,-p,q)$$, see Lemma 2.5. Therefore, since $$n^2_{\mathrm{top}}$$ is in direction $$M_1 \times M_2$$ and $$n^2_{\mathrm{bot}}$$ is in direction $$-M_1 \times (E_4-M_1)$$, we get $$n^2_{\mathrm{top}} = \frac{1}{\sqrt{s^2+2h^2}}(-h,-h,s)$$, and an elementary computation yields $$n^2_{\mathrm{bot}} = v/|v|$$ for $$v = (-h,-h,s) + (0, q s /p,0) $$. This implies $$n^2_{\mathrm{top}}$$ and $$n^2_{\mathrm{bot}}$$ are not parallel and therefore by (2.15) the incidence angle, denoted by $$\gamma ^*$$, is not zero.

To determine the sign of the non-zero incidence angle, we need to determine the sign of $(y^2_{\mathrm{top}}- y_{\mathrm{bot}}^2) \cdot (n^2_{\mathrm {top}} - n^2_{\mathrm{bot}}) = (y^2_{\mathrm{top}}- y_{\mathrm{bot}}^2) \cdot n^2_{\mathrm {top}} - (y^2_{\mathrm{top}}- y_{\mathrm{bot}}^2) \cdot n^2_{\mathrm{bot}}$. First note that $(y^2_{\mathrm{top}}- y_{\mathrm{bot}}^2) = M_2 - E_4 = (-p, s+p, h-q)$ which yields $(y^2_{\mathrm{top}}- y_{\mathrm{bot}}^2) \cdot n^2_{\mathrm {top}} = -\lambda qs$, with $\lambda = 1/\sqrt{s^2 + 2h^2}$ and $(y^2_{\mathrm{top}}- y_{\mathrm{bot}}^2) \cdot n^2_{\mathrm{bot}} = \mu qs^2/p$, with $\mu = 1/\vert v \vert$. Therefore, we obtain $(y^2_{\mathrm{top}}- y_{\mathrm{bot}}^2) \cdot (n^2_{\mathrm {top}} - n^2_{\mathrm{bot}}) = -\lambda qs - \mu qs^2/p < 0$. Hence, the incidence angle has a negative sign, see (2.15). Summarizing, we have shown that in D-tiles the angles are also zero along $d_1$ and lie in $\lbrace -\gamma ^*,\gamma ^* \rbrace$ along $d_2$.

*I-tiles.* It is obvious that for I-tiles, being combinations of Z- and D-tiles, we find that the incidence angles along $d_1$ are also 0 and along $d_2$ they lie in $\lbrace -\gamma ^*,0, \gamma ^* \rbrace$.

We close the proof by the observation that, due to the symmetries in Z-, D-, and I-tiles contained in ${\mathcal {A}}$, see (3.2), it is indeed elementary to check that $\gamma _2(s,t) = \gamma _2( s+1/2,t+1/2)$ for all $s,t\in \frac{1}{2}{\mathbb {Z}}$ with $s+t \in {\mathbb {Z}}+1/2$.

### Admissible Configurations and Ground States of the Energy

This subsection is devoted to the proof of Proposition 2.1.

#### Proof

*Step 1.* We start by introducing a specific *unit cell*: fix $x_0 \in {\mathbb {Z}}^2$ and denote the four neighbors of $x_0$ by $x_1 = x_0 + e_1$, $x_2 = x_0 + e_2$, $x_3 = x_0-e_1$, and $x_4 = x_0-e_2$. Given a deformation $y:\lbrace x_0,\ldots ,x_4\rbrace \rightarrow {\mathbb {R}}^3$, we define

$y_i = y(x_i)$ for $i=0,\ldots ,4$, and we let $y_{5} = y_{1}$. We introduce the *cell energy* by

A.7 $$\begin{aligned} E_{\mathrm{cell}}(y)= & {} \frac{1}{2}\sum _{i=1}^4 v_2(|y_i-y_0|) + \frac{1}{2}\sum _{i=1}^4 v_2(|y_i-y_{i+1}|) \nonumber \\&+ \sum _{i=1}^4 v_3(\theta _i) + v_3(\delta _{13}) + v_3(\delta _{24}), \end{aligned}$$

where $\theta _i = \measuredangle y_i\, y_{0} \, y_{i+1}$ for $i=1,\ldots ,4$, as well as $\delta _{13} = \measuredangle y_1 \, y_0\, y_3$ and $\delta _{24} = \measuredangle y_2 \, y_0 \, y_4$. The cell $(y_i)_{i=0}^4$ is called *optimal* if it minimizes (A.7).

Let us start by relating the cell energy to the configurational energy in (2.1). To this end, let $$y:{\mathbb {Z}}^2 \rightarrow {\mathbb {R}}^3$$ be a deformation, and for $$m \in {\mathbb {N}}$$ let $$Q_m$$ be the open square centered at 0 with sidelength 2*m*. For $$j \in {\mathbb {Z}}^2 \cap Q_m$$ we denote by $$y^j = \lbrace y_0^j,\ldots , y^j_4 \rbrace $$ the cell considered above for $$x_0 = j $$. Then, in view of (2.1), owing to the fact that bonds related to nearest-neighbors and next-to-nearest-neighbors are contained in two cells (apart from bonds intersecting $$\partial Q_m$$), whereas each bond angle is contained in exactly one cell, we find for every $$m \in {\mathbb {N}}$$ that

$$\begin{aligned} E(y,Q_m) =\sum _{j\in {\mathbb {Z}}^2 \cap Q_m} E_{\mathrm{cell}}(y^j) = (2m-1)^2 \frac{1}{\# ({\mathbb {Z}}^2 \cap Q_m)} \sum _{j\in {\mathbb {Z}}^2 \cap Q_m} E_{\mathrm{cell}}(y^j). \end{aligned}$$

Then, recalling the definition in (2.5) and by arguing as in [13, Proposition 2.1] we have that $y:{\mathbb {Z}}^2 \rightarrow {\mathbb {R}}^3$ is a ground state if and only if for each $x_0 \in {\mathbb {Z}}^2$ the corresponding cell $\lbrace y_0,\ldots , y_4\rbrace$ is optimal. Note that there exist admissible configurations consisting of optimal cells by Theorem 2.8, e.g., a tiling with only Z-tiles. Therefore, in the following it suffices to minimize the cell energy and to show that the unique minimizer is identified by having specific bond lengths and bond angles.

*Step 2.* Let $\{y_0, y_1, y_2, y_3, y_4\}$ be an optimal cell. We show that $\vert y_{j} - y_{0} \vert \in (1-\eta , 1+ \eta )$ as well as $\theta _j > \pi /2 - \eta$ for $j=1,\ldots ,4$. Assume first by contradiction that $\vert y_{j} - y_{0}\vert \le 1-\eta$ for some $j = 1,\dots ,4$. Then by using $v_2 \ge -1$, $v_3 \ge 0$, the fact that $v_2$ is decreasing on (0, 1), and (2.6) we get

$$\begin{aligned} E_{{\text {cell}}}(y)\ge & {} \frac{1}{2} \sum _{i=1}^4 v_2\left( \vert y_i - y_{0} \vert \right) + \frac{1}{2} \sum _{i=1}^4 v_2\left( \vert y_{i+1} - y_{i} \vert \right) \\\ge & {} \frac{1}{2} v_2(1-\eta ) + \frac{1}{2} \sum _{i\ne j} v_2 \left( \vert y_i - y_{0} \vert \right) + \frac{1}{2}\sum _{i=1}^4 v_2\left( \vert y_{i+1} - y_{i} \vert \right) \\\ge & {} \frac{1}{2} v_2(1-\eta ) - \frac{3}{2} -2 = \frac{1}{2} v_2(1-\eta ) -\frac{7}{2} \\&{\mathop {>}\limits ^{(2.6)}}&-2 + 2 v_2(\sqrt{2}) +4v_3(\pi /2) = E_{{\text {cell}}}(x_0, x_1,x_2,x_3,x_4). \end{aligned}$$

In the last equation, we have also used that $v_2(1) = -1$ and $v_3(\pi ) = 0$. This estimate contradicts the optimality of the cell.

In a similar fashion, we assume by contradiction that there exists some bond angle $\theta _j$, $j=1,\ldots ,4$, such that $\theta _j \le \pi /2 - \eta$. Then, by $v_2 \ge -1$, $v_3 \ge 0$, and (2.8) we have

$$\begin{aligned} E_{{\text {cell}}}(y)= & {} \frac{1}{2} \sum _{i=1}^4 v_2\left( \vert y_{i} - y_{0} \vert \right) + \frac{1}{2}\sum _{i=1}^4 v_2\left( \vert y_i - y_{i+1} \vert \right) \\&+ \sum _{i=1}^4 v_3(\theta _i) + v_3(\delta _{13}) + v_3(\delta _{24}) \\\ge & {} -4 + v_3(\theta _j) {\mathop {>}\limits ^{(2.8)}} -2 + 2 v_2(\sqrt{2}) +4v_3(\pi /2)\\= & {} E_{{\text {cell}}}(x_0, x_1,x_2,x_3,x_4), \end{aligned}$$

which is again in contradiction with the optimality of *y*.

We eventually show that for an optimal cell the bond lengths have to be less then $1+\eta$. Basic trigonometry together with the least size of the bond lengths and bond angles ensures that second-neighbor bonds have at least length

A.8 $$\begin{aligned} 2(1-\eta ) \sin (\pi /4 - \eta /2)= & {} 2(1-\eta )\frac{\sqrt{2}}{2} \left( \cos (\eta /2) - \frac{1}{2} \sin (\eta /2)\right) \nonumber \\> & {} \sqrt{2} (1-\eta )^2 > 1, \end{aligned}$$

where the last two inequalities hold for $\eta$ sufficiently small. Assume now that $|y_j - y_{0}| \ge 1 + \eta$ for some $j=1,\dots , 4$. Then, we get by $v_2 \ge -1$, $v_3 \ge 0$, the fact that $v_2$ increasing on $[1,\infty )$, and (2.7) that

$$\begin{aligned} E_{{\text {cell}}}(y)= & {} \frac{1}{2} \sum _{i=1}^4 v_2\left( \vert y_{i} - y_{0} \vert \right) + \frac{1}{2}\sum _{i=1}^4 v_2\left( \vert y_i - y_{i+1} \vert \right) \\&+ \sum _{i=1}^4 v_3(\theta _i) + v_3(\delta _{13}) + v_3(\delta _{24}) \\\ge & {} -\frac{3}{2} + \frac{1}{2} v_2(1+\eta ) + 2v_2(\sqrt{2}(1-\eta )^2) \\&{\mathop {>}\limits ^{(2.7)}}&-2 + 2 v_2(\sqrt{2}) +4v_3(\pi /2) = E_{{\text {cell}}}(x_0, x_1,x_2,x_3,x_4). \end{aligned}$$

The latter inequality once again contradicts optimality and we conclude that all first-neighbor bond lengths are at most $1+\eta$.

*Step 3.* To simplify notation, we denote the collection of angles by $\varvec{\theta } :=(\theta _i)_{i=1}^4 = (\theta _1,\dots ,\theta _4)$. We observe that $\delta _{24}$ can be written as a function of $\varvec{\theta }$ and $\delta _{13}$, i.e., $\delta _{24}= f( \varvec{\theta }, \delta _{13} )$, where the function *f* is explicitly given in Step 8, see (A.21). We will not need the exact form of this function, but only use that it is smooth for $\theta _i$ in a left neighborhood of $\pi /2$ and $\delta _{13}$ in a small interval left of $\pi$, see Step 8 below. Using Lemma 2.4 we find that in a cell with $\theta _1=\cdots =\theta _4 = \theta$ it holds that $\delta _{24} = f(\theta ,\ldots ,\theta ,\delta _{13}) = f_\theta (\delta _{13})$, where $f_\theta (\delta ) := 2\arccos \left( \cos \theta /\cos (\delta /2)\right)$. Note that $f_\theta$ has a unique fixed point $\delta _\theta := 2 \arccos (\sqrt{\cos \theta })$. We decompose the cell energy $E_{\mathrm{cell}}$ defined in (A.7) as

A.9 $$\begin{aligned} E_{\mathrm{cell}} (y) = \sum _{i=1}^4 F(\ell _i,\ell _{i+1}, \theta _i) + v_3(\delta _{13}) + v_3(f( \varvec{\theta }, \delta _{13} )), \end{aligned}$$

where $\ell _i := |y_i-y_0|$ for $i=1,\ldots ,4$ and

$$\begin{aligned} F(\ell _i,\ell _{i+1}, \theta _i):= & {} \frac{1}{4} v_2(\ell _i) + \frac{1}{4} v_2(\ell _{i + 1}) \\&+ \frac{1}{2} v_2 \big ((\ell _i^2 + \ell _{i + 1}^2 - 2 \ell _i\ell _{i + 1} \cos \theta _i)^{1/2}\big ) + v_3 (\theta _i). \end{aligned}$$

We have proved that, if $\{y_0,\dots ,y_4\}$ is optimal, first-neighbor bond lengths $\ell _i$ lie in $(1-\eta ,1 + \eta )$ and bond angles $\theta _i$ lie in $(\pi /2 - \eta , \pi ]$. Therefore, by using the convexity assumption (2.9) on *F* we find

A.10 $$\begin{aligned} \sum _{i=1}^4 F(\ell _i,\ell _{i+1}, \theta _i) \ge 4F({\bar{\ell }},{\bar{\ell }},{\bar{\theta }}), \end{aligned}$$

where

A.11 $$\begin{aligned} {\bar{\ell }}=\frac{1}{4}( \ell _1 + \dots + \ell _4), \quad {\bar{\theta }}=\frac{1}{4}( \theta _1 + \dots + \theta _4). \end{aligned}$$

Note that the inequality in (A.10) is strict whenever $\ell _i\not = {\bar{\ell }}$ or $\theta _i \not = {\bar{\theta }}$ for some $i=1,\dots ,4$.

*Step 4.* We check that the map $(\ell ,\theta ) \mapsto F(\ell ,\ell ,\theta )$ is minimized on $(1-\eta ,1+\eta ) \times (\pi /2-\eta ,\pi ]$ at some $\ell ^* \le 1$ and ${\theta }^*<\pi /2$. If we had $\ell ^* > 1$, one could reduce *F* by reducing $\ell$, noting that $v_2$ is increasing in $(1,\infty )$ and recalling (A.8). This, however, would contradict optimality. We now exclude $\theta ^* \ge \pi /2$. Indeed, in this case we could decrease $\theta ^*$ by $0<{\tilde{\theta }} \ll 1$ and by a Taylor expansion we would get that *F* changes to first order by

$$\begin{aligned} -v_3'(\theta ^*){\tilde{\theta }} - v_2'\big (\sqrt{2}\ell \sqrt{1-\cos \theta ^*}\big ) \frac{\ell }{2\sqrt{2}} \frac{\sin \theta ^* \, {\tilde{\theta }}}{\sqrt{1-\cos \theta ^*}}. \end{aligned}$$

By $\ell \in (1-\eta ,1]$ and (2.11) we get that the above term is negative, which contradicts minimality.

*Step 5.* Next, we show that for ${\bar{\theta }}$ defined in (A.11) it holds that ${\bar{\theta }} \le \pi /4 + \theta ^*/2$. We also establish a bound from below on $\delta _{13}$ and $\delta _{24}$. The argument is based on the observation that by (A.9), (A.10), and the definition of $\delta _\theta$ we find that $4F(\ell ,\ell ,{\theta }) + 2v_3(\delta _{\theta })$ is an upper bound for the minimal cell energy for $(\ell ,\theta ) \in (1-\eta ,1+\eta ) \times (\pi /2-\eta ,\pi ]$. By definition we have $\delta _\theta \rightarrow \pi$ as $\theta \rightarrow \pi /2$. Thus, in view of (2.10), the monotonicity of $v_3$, and $\theta ^* \ge \pi /2-\eta$, we can choose $\eta$ sufficiently small depending on $v_3$ and find $\lambda >0$ small such that $|v_3| \le \varepsilon$ on $[\pi -\lambda ,\pi ]$, $|v_3'| \le \varepsilon$ on $[\pi - 2\lambda , \pi ]$, and

A.12 $$\begin{aligned} v_3(\delta )> 2\varepsilon > 2v_3(\delta _{\theta ^*}) \quad \text { for }\, \delta \le \pi - \lambda . \end{aligned}$$

We also suppose that $\varepsilon$ is chosen small enough depending on $v_2$, $v_3$, and $\theta ^*$ such that

A.13 $$\begin{aligned} F(\ell ,\ell ,\theta )> F(\ell ^*,\ell ^*,\theta ^*) + 2\varepsilon \ \ \ \text {for} \ \theta > \frac{\pi }{4} + \frac{\theta ^*}{2} \text { and}\, \ell \in (1-\eta ,1+\eta ). \end{aligned}$$

Now, we can suppose $\delta _{13},\delta _{24} \ge \pi -\lambda$ (recall that $\delta _{24} = f(\varvec{\theta },\delta _{13})$) since otherwise we get

$$\begin{aligned} {E_{\mathrm{cell}} (y) > 4F( {\bar{\ell }},{\bar{\ell }}, {\bar{\theta }}) + 2v_3(\delta _{\theta ^*}) \ge 4F( \ell ^*,\ell ^*, {\theta }^*) + 2v_3(\delta _{\theta ^*})} \end{aligned}$$

by using (A.9), (A.10), and (A.12), which contradicts minimality. In a similar fashion, we can suppose that ${\bar{\theta }}$ in (A.11) satisfies ${\bar{\theta }} \le \frac{\pi }{4} + \frac{\theta ^*}{2}$ as otherwise $E_{\mathrm{cell}} (y) > 4F({\ell }^*,{\ell }^*,{\theta }^*) + 2v_3(\delta _{\theta ^*})$ follows using (A.9), (A.10), (A.12), and (A.13).

*Step 6.* We are left with the case $\delta _{13} \ge \pi -\lambda$ and $\theta _1 + \ldots + \theta _4 = 4 {\bar{\theta }} \le \pi + 2\theta ^* < 2\pi$. In this step, we show that

A.14 $$\begin{aligned} E_{\mathrm{cell}} (y) \ge 4F({\bar{\ell }},{\bar{\ell }},{\bar{\theta }}) + v_3(\delta _{13}) + v_3(f_{{\bar{\theta }}}(\delta _{13})) \end{aligned}$$

with equality only if $\ell _i = {\bar{\ell }}$ and $\theta _i = {\bar{\theta }}$ for $i=1,\dots ,4$.

We start by noticing that $\theta _1 + \ldots + \theta _4 <2\pi$ and $\theta _i > \pi /2 - \eta$ for $i=1,\ldots ,4$ imply $\theta _i < \pi /2 + 3\eta$ for $i=1,\ldots ,4$. Therefore, the convexity estimate in (A.10) can be improved by using the strong convexity assumption (2.9) on *F*, and we find

A.15 $$\begin{aligned} \sum _{i=1}^4 F(\ell _i,\ell _{i+1}, \theta _i) \ge 4F({\bar{\ell }},{\bar{\ell }},{\bar{\theta }}) + \alpha \sum _{i=1}^4 \vert \theta _i - {\bar{\theta }} \vert ^2 \end{aligned}$$

for some $\alpha >0$. Moreover, a simple geometric argument shows that $\delta _{13} = \pi$ implies $\theta _1 + \ldots + \theta _4 = 2\pi$, see Fig. 14. Therefore, by a continuity argument and $\theta _1 + \ldots + \theta _4 \le \pi + 2\theta ^*$ we get that $\delta _{13} \le \delta ^*$ for some $\delta ^* < \pi$ only depending on $\theta ^*$. Consequently, we need to consider the case that $\delta _{13} \in [\pi - \lambda ,\delta ^*]$ and $\theta _i\in (\frac{\pi }{2}-\eta ,\frac{\pi }{2}+3\eta )$.

If $\alpha \sum _{i = 1}^4 |\theta _i - {{\bar{\theta }}}|^2 \ge 2\varepsilon$, by (A.9), (A.12), and (A.15) we obtain a contradiction to minimality as $v_3(\delta _{13}) + v_3(f(\varvec{\theta },\delta _{13})) + \alpha \sum _{i = 1}^4 |\theta _i - {{\bar{\theta }}}|^2 \ge 2\varepsilon > 2v_3(\delta _{{\theta }^*})$.

If $\alpha \sum _{i = 1}^4 |\theta _i - {{\bar{\theta }}}|^2 < 2\varepsilon$, we now show that $f_{{{\bar{\theta }}}}(\delta _{13})$ cannot be too far away from $f(\varvec{\theta },\delta _{13})$. Eventually, this will allow us to deduce (A.14). By choosing $\varepsilon$ small enough and recalling that ${{\bar{\theta }}}<\pi /2$, we get that $\theta _i \le \pi /2$ for $i=1,\ldots ,4$. By Taylor’s Theorem there exists ${{\varvec{z}}}\in \{ t\varvec{\theta } + (1-t) \varvec{{\bar{\theta }}} \, : \, t \in [0,1] \}$, where $\bar{\varvec{\theta }} = ({\bar{\theta }}, \dots , {\bar{\theta }})$, such that

A.16 $$\begin{aligned} f(\varvec{\theta },\delta _{13}) - f_{{\bar{\theta }}}(\delta _{13})= & {} f(\varvec{\theta },\delta _{13}) - f(\varvec{{\bar{\theta }}},\delta _{13}) \nonumber \\= & {} \nabla _{\varvec{\theta }} f(\varvec{{\bar{\theta }}},\delta _{13}) (\varvec{\theta } - \varvec{{\bar{\theta }}}) + \dfrac{1}{2} (\varvec{\theta } - \varvec{{\bar{\theta }}}) \nabla _{\varvec{\theta }}^2 f({{\varvec{z}}}, \delta _{13}) (\varvec{\theta } - \varvec{{\bar{\theta }}}) \nonumber \\= & {} \dfrac{1}{2} (\varvec{\theta } - \varvec{{\bar{\theta }}}) \nabla _{\varvec{\theta }}^2 f({{\varvec{z}}}, \delta _{13}) (\varvec{\theta } - \varvec{{\bar{\theta }}}) \le \frac{\lambda _{\max }}{2} | \varvec{\theta } - \varvec{{\bar{\theta }}} |^2 \nonumber \\= & {} \frac{\lambda _{\max }}{2} \sum _{i=1}^4 \vert \theta _i -{\bar{\theta }} \vert ^2, \end{aligned}$$

where we used that $\tfrac{\partial }{\partial \theta _i} f(\varvec{{\bar{\theta }}}, \delta _{13}) = \tfrac{\partial }{\partial \theta _j} f(\varvec{{\bar{\theta }}}, \delta _{13})$ and thus

$$\begin{aligned}{ \nabla _{\varvec{\theta }} f(\varvec{{\bar{\theta }}}, \delta _{13}) \cdot (\varvec{\theta } - \varvec{{\bar{\theta }}}) = \tfrac{\partial }{\partial \theta _1} f(\varvec{{\bar{\theta }}}, \delta _{13}) \left( \sum _i \theta _i - \sum _i {\bar{\theta }} \right) = 0.} \end{aligned}$$

In (A.16) we denoted the largest eigenvalue of the Hessian $\nabla _{\varvec{\theta }}^2 f({{\varvec{z}}},\delta _{13})$ with $\lambda _{\max }$. Using the Gershgorin circle theorem, we find $\vert \lambda _{\max } \vert \le 4 c_f$ where we use that *f* is smooth for $\theta _i \in I:=[\frac{\pi }{2}-\eta ,\frac{\pi }{2}]$ and $\delta _{13} \in [\pi - \lambda ,\delta ^*]$, and define

$$\begin{aligned} c_{f} := \max _{i,j = 1,\dots ,4} \sup _{\theta _i \in I} \sup _{\delta _{13} \in [\pi - \lambda , \delta ^*]} \left| \frac{\partial ^2}{\partial \theta _i \partial \theta _j} f (\varvec{\theta },\delta _{13}) \right| < \infty . \end{aligned}$$

The proof of the smoothness of *f* is deferred to the end of the proof in Step 8. Therefore, we obtained

A.17 $$\begin{aligned} \vert f(\varvec{\theta },\delta _{13}) - f_{{\bar{\theta }}}(\delta _{13}) \vert \le 2 c_f \sum _{i=1}^4 \vert \theta _i -{\bar{\theta }} \vert ^2. \end{aligned}$$

For $\varepsilon$ small enough such that $4 c_f \varepsilon \le \alpha \lambda$, due to $\alpha \sum _{i = 1}^4 |\theta _i - {{\bar{\theta }}}|^2 < 2\varepsilon$, we have

$$\begin{aligned} \vert f(\varvec{\theta },\delta _{13}) - f_{{\bar{\theta }}}(\delta _{13}) \vert \le 2 c_f \sum _{i=1}^4 \vert \theta _i -{\bar{\theta }} \vert ^2 \le \frac{\alpha \lambda }{2 \varepsilon } \sum _{i=1}^4 \vert \theta _i -{\bar{\theta }} \vert ^2 \le \lambda . \end{aligned}$$

Hence $f_{{{\bar{\theta }}}}(\delta _{13}) \ge \pi - 2\lambda$ as $f(\varvec{\theta },\delta _{13}) \ge \pi -\lambda$ by Step 5. Therefore, as $|v_3'| \le \varepsilon$ on $[\pi -2\lambda ,\pi ]$ and $\lambda >0$ small, we obtain by (A.17)

A.18 $$\begin{aligned} \vert v_3(f(\varvec{\theta },\delta _{13})) - v_3(f_{{\bar{\theta }}}(\delta _{13})) \vert\le & {} \vert f(\varvec{\theta }, \delta _{13}) - f_{{\bar{\theta }}}(\delta _{13})\vert \sup _{[\pi -2\lambda , \pi ]} \vert v'_3\vert \nonumber \\\le & {} 2 c_f \varepsilon \sum _{i=1}^4 \vert \theta _i -{\bar{\theta }} \vert ^2 \le \frac{\alpha }{2} \sum _{i=1}^4 \vert \theta _i -{\bar{\theta }} \vert ^2. \end{aligned}$$

Consequently, (A.14) holds by applying (A.15) and (A.18) to (A.9).

*Step 7.* We now conclude the proof by showing

A.19 $$\begin{aligned} E_{\mathrm{cell}} (y) \ge 4 \left( \frac{1}{2} v_2({\bar{\ell }}) + \frac{1}{2}v_2 ( \sqrt{2} {\bar{\ell }} (1 -\cos {\bar{\theta }})^{1/2}) + v_3 ({\bar{\theta }}) \right) + 2v_3(\delta _{{\bar{\theta }}}), \end{aligned}$$

where $\delta _{{\bar{\theta }}} =2 \arccos (\sqrt{\cos {\bar{\theta }}})$, and that equality holds only if $\ell _i = {\bar{\ell }}$ and $\theta _i = {\bar{\theta }}$ for $i=1,\dots ,4$. To this end, we further estimate (A.14) by claiming

A.20 $$\begin{aligned} g(\delta _{13}) := v_3(\delta _{13}) + v_3(f_{{\bar{\theta }}}(\delta _{13})) \ge 2v_3(\delta _{{\bar{\theta }}}), \end{aligned}$$

with equality if and only if $\delta _{13} = f_{{\bar{\theta }}}(\delta _{13})$. Computing $g'(\delta ) = v_3'(\delta ) + v_3'(f_{{{\bar{\theta }}}}(\delta )) \, f'_{{{\bar{\theta }}}}(\delta )$ shows that $g'(\delta _{{\bar{\theta }}}) = 0$, because $f'_{{{\bar{\theta }}}}(\delta _{{\bar{\theta }}}) = -1$ and $\delta _{{\bar{\theta }}} = f_{{\bar{\theta }}}(\delta _{{\bar{\theta }}})$. Moreover, we calculate $g''(\delta ) = v_3''(\delta ) + v_3''(f_{{{\bar{\theta }}}}(\delta )) \, (f'_{{{\bar{\theta }}}}(\delta ))^2 + v_3'(f_{{{\bar{\theta }}}}(\delta )) \, f''_{{{\bar{\theta }}}}(\delta )>0$, where we used the monotonicity and strict convexity of $v_3$ and the concavity of $f_{{{\bar{\theta }}}}$, which follows from an elementary computation. This indeed implies (A.20).

This, along with (A.14), implies that (A.19) holds, with equality only if all bonds of an optimal cell have length ${\bar{\ell }}$, all angles have amplitude ${\bar{\theta }}$, and $\delta _{13} = \delta _{24} = \delta _{{\bar{\theta }}}$. Clearly, for an optimal cell, ${\bar{\ell }}$ and ${\bar{\theta }}$ are given uniquely. We finally observe that ${\bar{\ell }} \le 1$ and ${\bar{\theta }} <\pi /2$. For ${\bar{\ell }}$, this follows from the fact that $\ell ^* \le 1$, as shown in Step 4, and ${\bar{\theta }}<\pi /2$ has been checked in Step 5.

*Step 8.* Let us conclude by collecting some remarks on the function $f(\varvec{\theta },\delta _{13})$ used throughout the proof. If $\delta _{13} = \pi$, we have that $\theta _1 + \theta _2 = \theta _3 + \theta _4 = \delta _{13} = \pi$. Therefore, $\delta _{24}$ can be chosen arbitrarily in $[0,\pi ]$, see Fig. 14, and $f(\cdot ,\pi )$ is hence not defined. For $\delta _{13} < \pi$, the definition is given by

A.21 $$\begin{aligned} f(\varvec{\theta } , \delta _{13})= & {} \arccos \left\{ \cos \theta _1 \, \cos \theta _4 +\frac{(\cos \theta _2 - \cos \delta _{13} \cos \theta _1)(\cos \theta _3 - \cos \delta _{13}\cos \theta _4)}{\sin ^2\delta _{13}} \right. \nonumber \\&\left. - \sqrt{1-\cos ^2\theta _1 - \frac{(\cos \theta _2 - \cos \delta _{13} \cos \theta _1)^2}{\sin ^2\delta _{13}}} \sqrt{1-\cos ^2\theta _4 -\frac{(\cos \theta _3 - \cos \delta _{13} \cos \theta _4)^2}{\sin ^2\delta _{13}}} \right\} ,\nonumber \\ \end{aligned}$$

which can be derived by elementary, yet tedious, trigonometry. Let us now check that *f* is smooth for all $\delta _{13} \in [\pi -\lambda ,\delta ^*]$ and $\varvec{\theta } \in [\pi /2-\eta ,\pi ]^4$ such that $f(\varvec{\theta } , \delta _{13}) = \delta _{24} \in [\pi -\lambda ,\delta ^*]$ which we need in Step 6 of the proof. First, since $\delta _{24} \in [\pi -\lambda ,\delta ^*]$ the expression inside of $\arccos$ is bounded away from $-1$ and 1. As $\delta _{13} \in [\pi -\lambda ,\delta ^*]$, $\sin \delta _{13}$ is bounded away from 0. Thus, it suffices to check that the expressions inside the square roots are bounded away from 0. Indeed, $\varvec{\theta } \in [\pi /2-\eta ,\pi ]^4$ implies $\cos \theta _1 \, \cos \theta _4 \rightarrow 0$ as $\eta \rightarrow 0$ and $(\cos \theta _2 - \cos \delta _{13} \cos \theta _1)(\cos \theta _3 - \cos \delta _{13}\cos \theta _4)/\sin ^2\delta _{13} \ge 0$. As $\cos (f(\varvec{\theta } , \delta _{13}))$ lies in a neighborhood of $-1$, this is indeed only possible if the value of each of the square roots is close to 1. $\square$
